# Supplementary material for: Aquaculture at the crossroads of global warming and antimicrobial resistance
Source: Nat Commun. 2020 Apr 20;11:1870. doi: 10.1038/s41467-020-15735-6 (PMC7170852; doi:10.1038/s41467-020-15735-6)
Supplement: Supplementary file 1 — Supplementary Information [file 41467_2020_15735_MOESM1_ESM.pdf]

Aquaculture at the crossroads of global warming and antimicrobial resistance

Reverter et al.

Supplementary figures

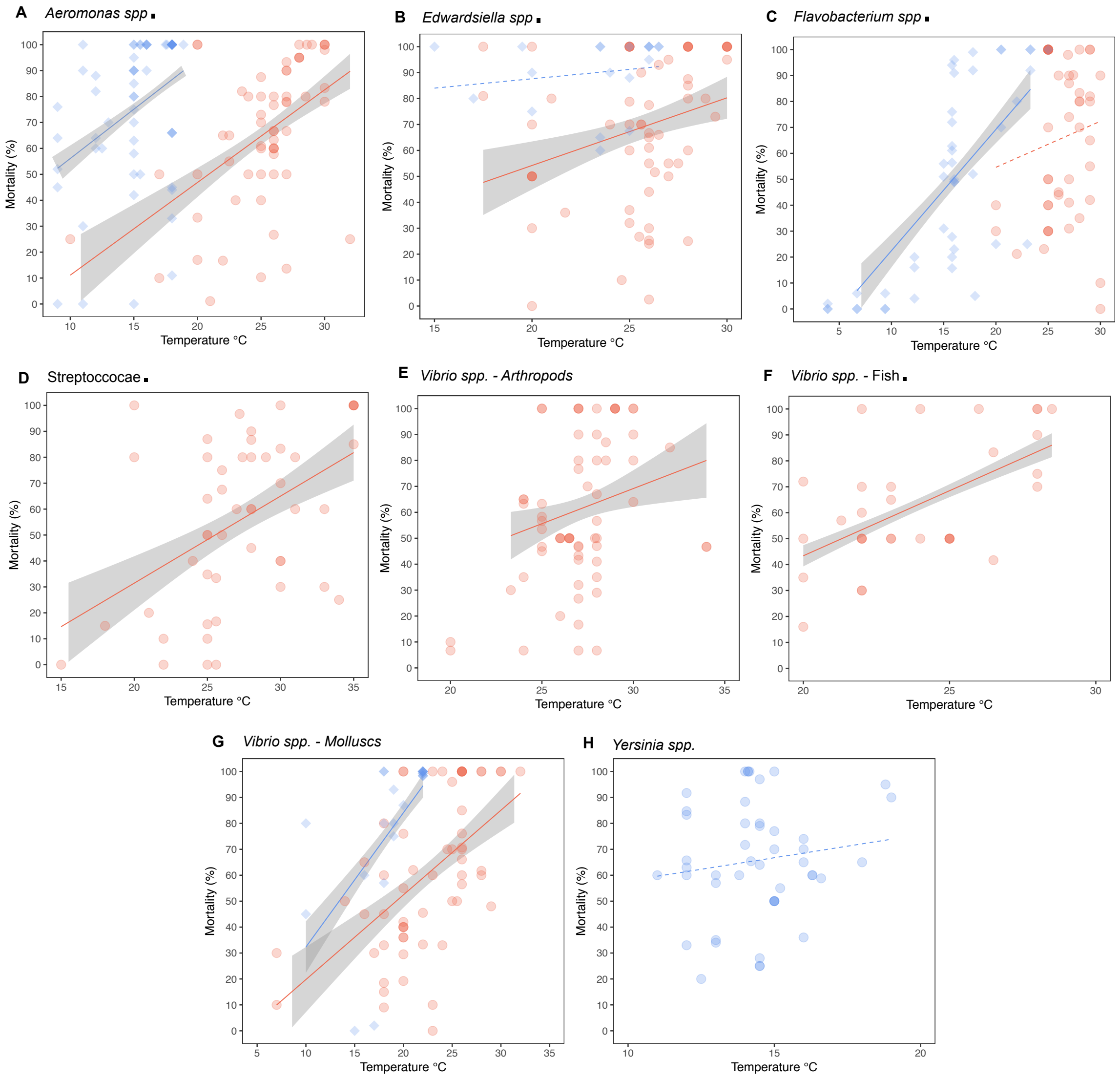

Supplementary figure 1. Predicted changes in mortality (%) of reared aquatic animals infected by bacterial A) *Aeromonas spp., B) Edwardsiella spp., C) F. columnare, D)* Streptoccocae (*Streptococcus spp., Lactococcus spp.), E-G) Vibrio spp.* and H) *Y. ruckeri* related to temperature (°C). Red indicates tropical and subtropical host species, blue indicates temperate host species. Dots represent the raw data and smooth lines the linear mixed model predictions (Supplementary table 15). Dashed lines indicate non-significant models. Shadow area represent SE.

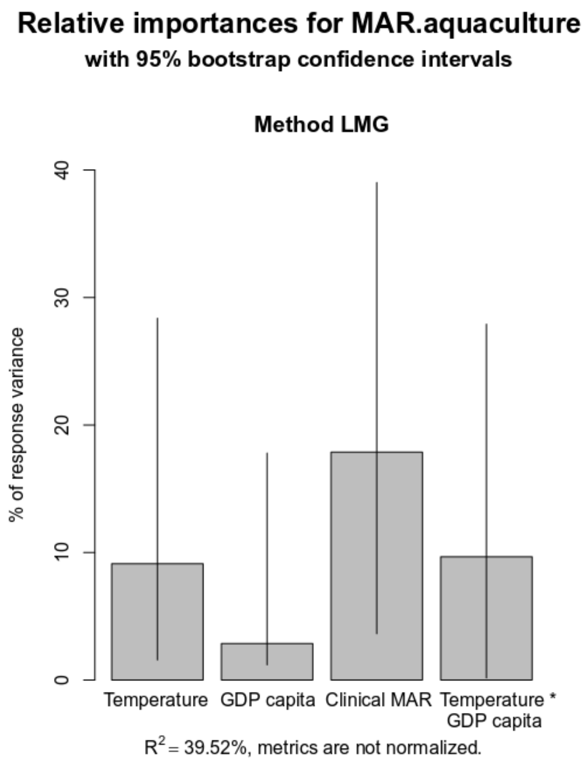

Supplementary figure 2. Relative importance of regressors from the multiple regression model (aquaculture MAR ~ Temperature + GDP capita + Clinical MAR + Temperature*GDP capita) with 95% bootstrap confidence intervals (1000 permutations) assessed by the Lindeman Merenda and Gold (LMG) metrics of the relaimpo R package.

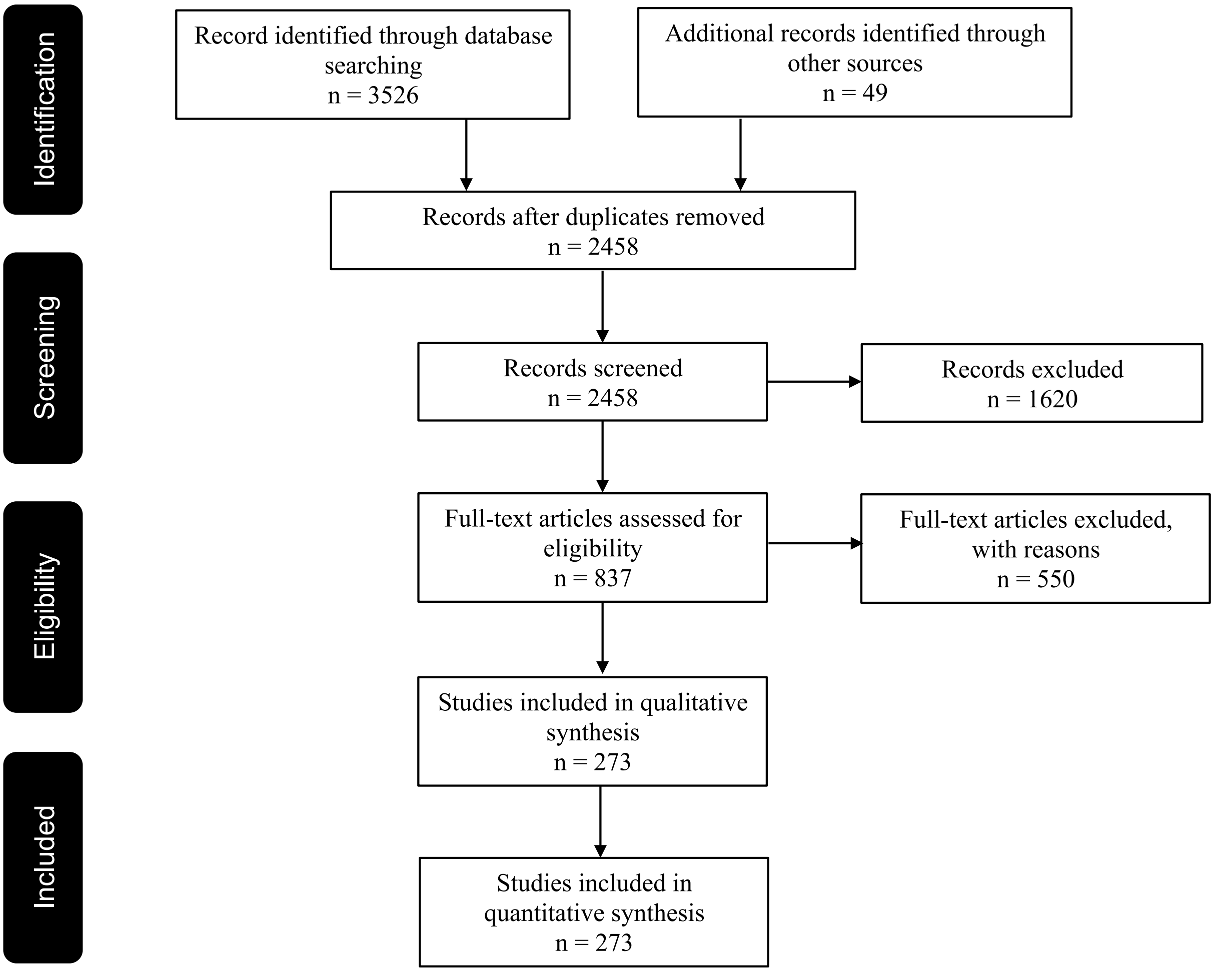

Supplementary figure 3. PRISMA diagram showing the process for locating and including studies in the meta-analysis investigating mortality of aquatic organisms and temperature.

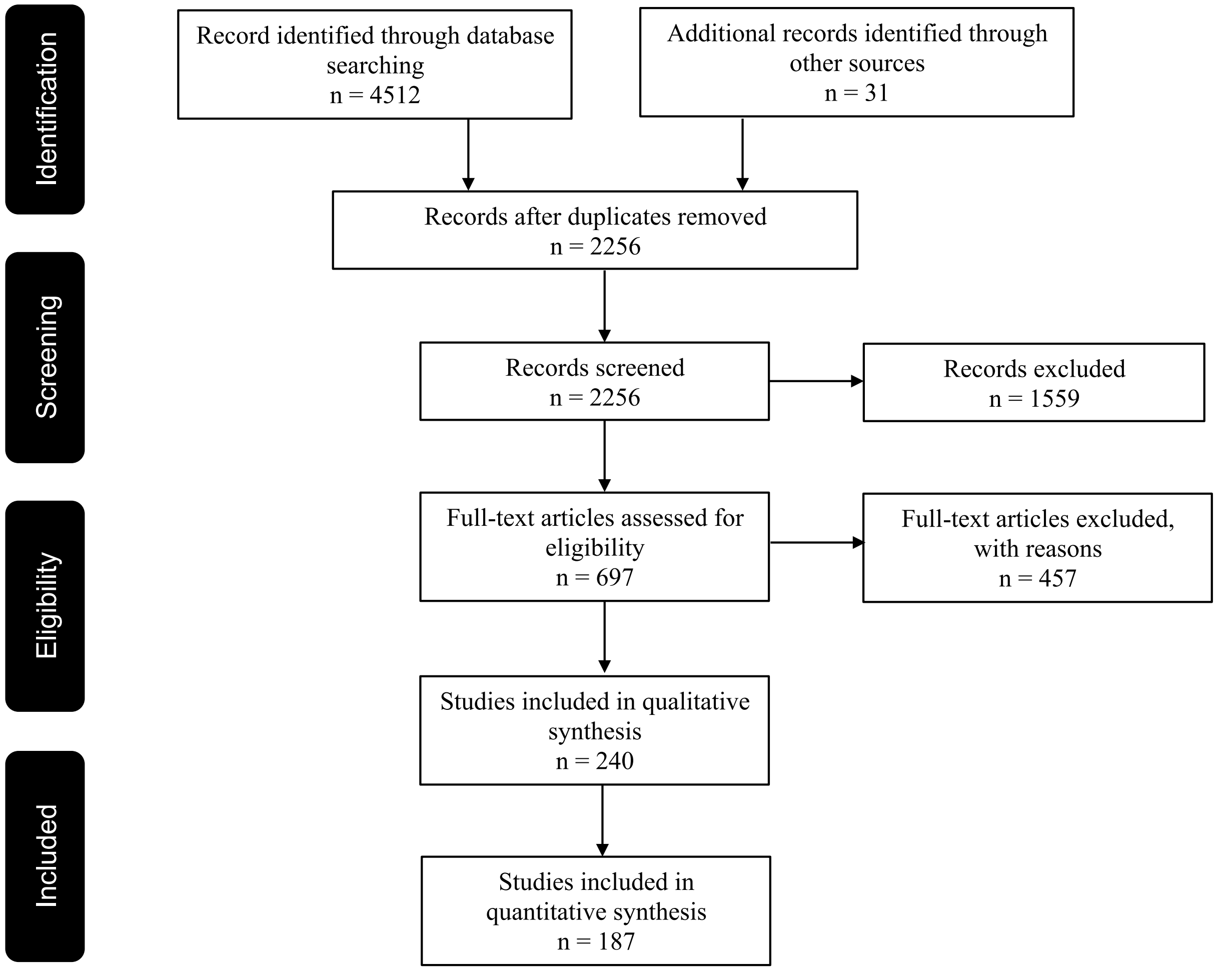

Supplementary figure 4. PRISMA diagram showing the proccess for locating and including studies in the meta-analysis investigating antibiotic resistance of aquaculture-related bacteria.

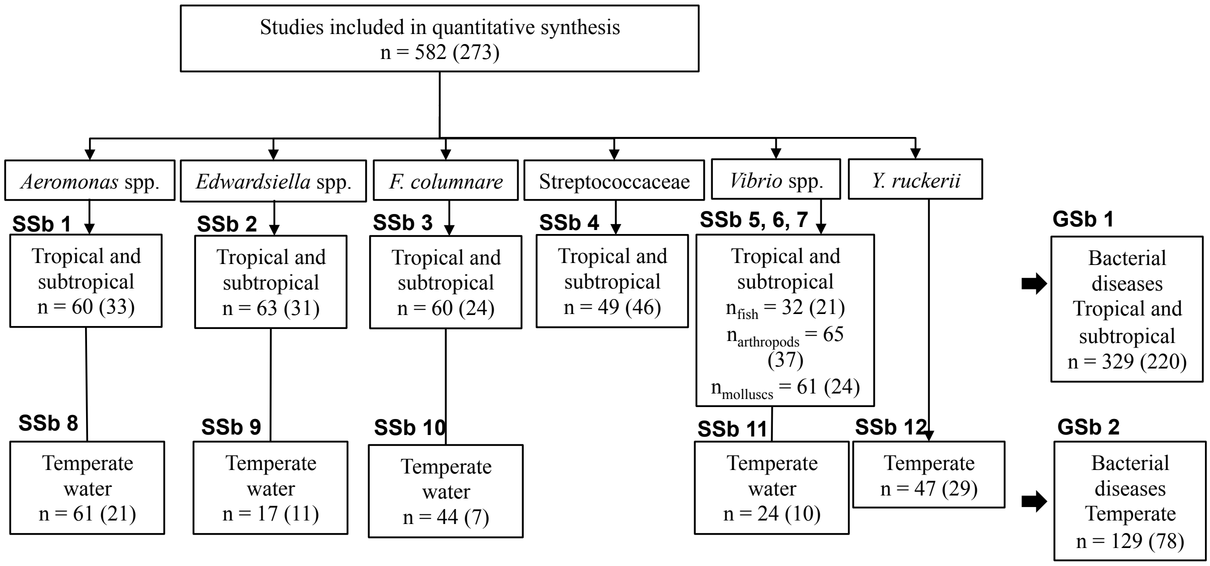

Supplementary figure 5. Diagram showing the different data subsets investigating mortality of aquatic organisms and temperature.

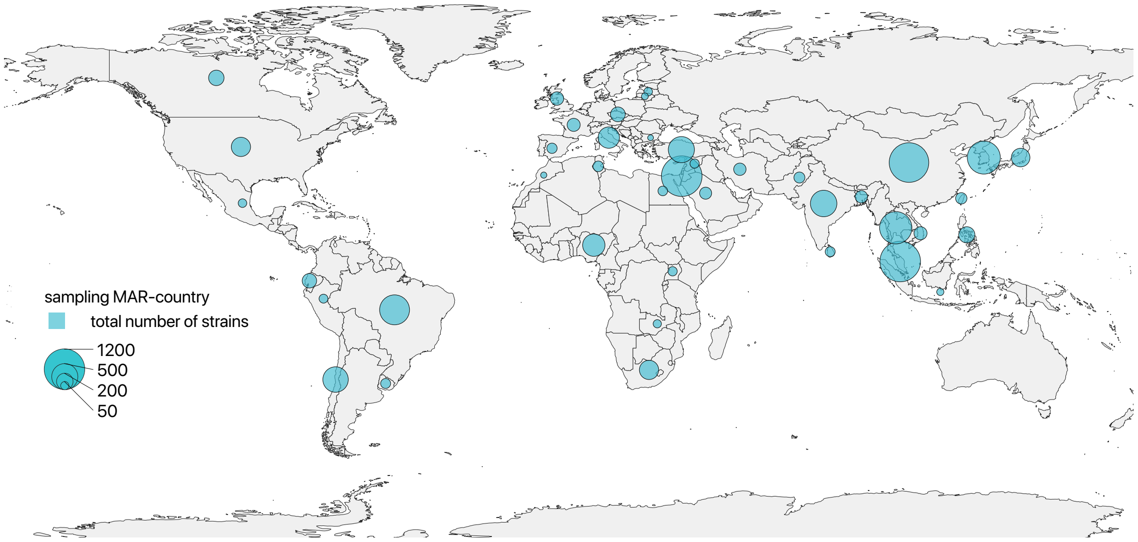

Supplementary figure 6. Global sampling of aquaculture-related antimicrobial resistant bacteria.

Supplementary Table 1. Warm-water fish infected with *Aeromonas.* Specific subset 1 (SSb 1).

Model ranking	Model	df	AICc	ΔAICc	w_i
Selection of random effects					
1	Mortality ~ T + (1 | Study)	4	551.2	0.00	0.466
2	Mortality ~ T + (1 | host family) + (1 | Study)	5	552.0	0.86	0.304
3	Mortality ~ T + (1 | pathogen species) + (1 | Study)	5	553.6	2.38	0.142
4	Mortality ~ T + (1 | pathogen species) + (1 | host family) + (1 | Study)	6	554.5	3.33	0.088
Selection of fixed effects (linear regression model)					
**1**	**Mortality ~ T + (1 | Study)**	**4**	**551.2**	**0.00**	**0.534**
2	Mortality ~ T + log(dose) + (1 | Study)	5	553.2	2.02	0.195
3	Mortality ~ T + mode of infection + (1 | Study)	6	554.3	3.15	0.110
4	Mortality ~ T + life stage + (1 | Study)	6	555.3	4.14	0.067
5	Mortality ~ T + mode of infection + log(dose) + (1 | Study)	7	556.5	5.26	0.038
6	Mortality ~ T + log(dose) + life stage + (1 | host Study)	7	557.2	6.01	0.026
7	Mortality ~ T + mode of infection*log(dose) + (1 | Study)	8	558.8	7.64	0.012
8	Mortality ~ T + mode of infection + life stage + (1 | Study)	8	558.9	7.75	0.0101
9	Mortality ~ T + life stage + mode of infection + log(dose) + (1 | Study)	9	561.0	9.78	0.004
10	Mortality ~ T + life stage + mode of infection*log(dose) + (1 | Study)	10	563.6	12.46	0.001

Supplementary Table 2. Warm-water fish infected with *Edwardsiella*. Specific subset 2 (SSb2).

Model ranking	Model	df	AICc	ΔAICc	w_i
Selection of random effects					
1	Mortality ~ T + (1 | host family) + (1 | Study)	5	590.5	0.00	0.583
2	Mortality ~ T + (1 | Study)	4	592.8	2.27	0.187
3	Mortality ~ T + (1 | host family) + (1 | pathogen species) + (1 | Study)	6	592.9	2.45	0.172
4	Mortality ~ T + (1 | pathogen species) + (1 | Study)	5	595.1	4.63	0.058
Selection of fixed effects (linear mixed model)					
**1**	**Mortality ~ T + mode of infection*log(dose) + (1 | host family) + (1 | Study)**	**8**	**581.4**	**0.00**	**0.287**
2	Mortality ~ T + mode of infection*log(dose) life stage + (1 | host family) + (1 | Study)	9	581.9	0.45	0.229
3	Mortality ~ T + mode of infection + (1 | host family) + (1 | Study)	6	582.4	0.97	0.177
4	Mortality ~ T + mode of infection + life stage + (1 | host family) + (1 | Study)	7	583.2	1.81	0.116
5	Mortality ~ T + mode of infection + log(dose) + (1 | host family) + (1 | Study)	7	583.4	1.93	0.110
6	Mortality ~ T + mode of infection + log(dose) + life stage + (1 | host family) + (1 | Study)	8	584.5	3.06	0.062
7	Mortality ~ T + log(dose) + (1 | host family) + (1 | Study)	6	588.7	7.26	0.008
8	Mortality ~ T + log(dose) + life stage + (1 | host family) + (1 | Study)	7	589.2	7.80	0.006
9	Mortality ~ T + life stage + (1 | host family) + (1 | Study)	6	590.5	9.02	0.003
10	Mortality ~ T + (1 | host family) + (1 | Study)	5	590.5	9.06	0.003

Supplementary Table 3. Warm-water fish infected with *Flavobacterium columnare*. Specific subset 3 (SSb3).

Model ranking	Model	df	AICc	ΔAICc	w_i
Selection of random effects					
1	Mortality ~ T + (1 | Study)	4	469.9	0.00	0.751
2	Mortality ~ T + (1 | host family) + (1 | Study)	5	472.1	2.21	0.249
Selection of fixed effects (linear regression model)					
**1**	**Mortality ~ T + log(dose)** + (1 | Study)	**5**	**469.6**	**0.00**	**0.313**
2	Mortality ~ T + (1 | Study)	4	469.9	0.25	0.276
3	Mortality ~ T + mode of infection + (1 | Study)	5	472.2	2.55	0.087
4	Mortality ~ T + mode of infection + log(dose) + (1 | Study)	6	472.2	2.61	0.085
5	Mortality ~ T + life stage + log(dose) + (1 | Study)	6	472.2	2.62	0.085
6	Mortality ~ T + life stage + (1 | Study)	5	472.4	2.74	0.079
7	Mortality ~ T + mode of infection + life stage + (1 | Study)	6	474.8	5.17	0.024
8	Mortality ~ T + mode of infection*log(dose) + (1 | Study)	7	474.9	5.22	0.023
9	Mortality ~ T + mode of infection + log(dose) + life stage + (1 | Study)	7	475.0	5.36	0.021
10	Mortality ~ T + mode of infection*log(dose) + life stage + (1 | Study)	8	477.7	8.08	0.006

Supplementary Table 4. Warm-water fish infected with Streptococcae bacteria (*Streptococcus spp.*, *Lactococcus spp.*). Specific subset 4 (SSb 4).

Model ranking	Model	df	AICc	ΔAICc	w_i
Selection of random effects					
1	Mortality ~ T + (1 | Study)	4	460.6	0.00	0.585
2	Mortality ~ T + (1 | host family) + (1 | Study)	5	462.9	2.22	0.193
3	Mortality ~ T + (1 | pathogen species) + (1 | Study)	5	463.1	2.49	0.169
4	Mortality ~ T + (1 | pathogen species) + (1 | host family) + (1 | Study)	6	465.5	4.82	0.053
Selection of fixed effects (linear mixed model)					
**1**	**Mortality ~ T + mode of infection + (1 | Study)**	**5**	**453.1**	**0.00**	**0.468**
2	Mortality ~ T + mode of infection + log(dose) + (1 | Study)	6	454.0	0.90	0.298
3	Mortality ~ T + mode of infection*log(dose + (1 | Study)	7	456.1	2.96	0.106
4	Mortality ~ T + mode of infection + life stage + (1 | Study)	7	457.0	3.91	0.066
5	Mortality ~ T + mode of infection + log(dose) + life stage + (1 | Study)	8	458.4	5.32	0.033
6	Mortality ~ T + (1 | Study)	4	460.6	7.52	0.011
7	Mortality ~ T + mode of infection*log(dose) + life stage + (1 | Study)	9	461.0	7.86	0.009
8	Mortality ~ T + log(dose) + (1 | Study)	5	462.5	9.38	0.004
9	Mortality ~ T + life stage + (1 | Study)	6	463.3	10.17	0.003
10	Mortality ~ T + life stage + log(dose) + (1 | Study)	7	465.5	12.42	0.001

Supplementary Table 5. Warm-water fish infected with *Vibrio*. Specific subset 5 (SSb5). Study was initially considered as a random effect, but it was detected as a singular variable (variance of random effect of 0), and therefore it was deleted to avoid overfitted models.

Model ranking	Model	df	AICc	ΔAICc	w_i
Selection of random effects					
1	Mortality ~ T	3	286.0	0.00	0.623
2	Mortality ~ T + (1 | pathogen species)	4	288.7	2.62	0.168
3	Mortality ~ T + (1 | host family)	4	288.7	2.62	0.168
4	Mortality ~ T + (1 | host family) + (1 | pathogen species)	5	291.5	5.45	0.041
Selection of fixed effects (linear regression model)					
**1**	**Mortality ~ T + Mode of infection**	**4**	**285.0**	**0.00**	**0.211**
2	Mortality ~ T + log(dose)	4	285.8	0.77	0.143
3	Mortality ~ T + host life stage + log(dose)	6	286.0	0.97	0.130
4	Mortality ~ T	3	286.0	0.98	0.129
5	Mortality ~ T + mode of infection + log(dose)	5	286.2	1.15	0.119
6	Mortality ~ T + life stage	5	286.7	1.68	0.091
7	Mortality ~ T + mode of infection*log(dose) + life stage	8	287.5	2.44	0.062
8	Mortality ~ T + mode of infection*log(dose)	6	287.5	2.48	0.061
9	Mortality ~ T + mode of infection + life stage	6	289.0	3.96	0.029
10	Mortality ~ T + mode of infection + log(dose) + life stage	7	289.3	4.22	0.025

Supplementary Table 6. Warm-water arthropods infected with *Vibrio*. Specific subset 6 (SSb6).

Model ranking	Model	df	AICc	ΔAICc	w_i
Selection of random effects					
1	Mortality ~ T + (1 | Study)	4	595.3	0.00	0.529
2	Mortality ~ T + (1 | pathogen species) + (1 | Study)	5	596.9	1.60	0.238
3	Mortality ~ T + (1 | host family) + (1 | Study)	5	597.7	2.35	0.163
4	Mortality ~ T + (1 | host family) + (1 | pathogen species) + (1 | Study)	6	599.3	4.03	0.070
Selection of fixed effects (linear mixed model)					
1	**Mortality ~ T + mode of infection + (1 | Study)**	**5**	**594.5**	**0.00**	**0.355**
2	Mortality ~ T + (1 | Study)	4	595.3	0.83	0.234
3	Mortality ~ T mode of infection*log(dose) + (1 | Study)	6	596.6	1.78	0.146
4	Mortality ~ T + log(dose) + (1 | Study)	5	596.6	2.10	0.124
5	Mortality ~ T + mode of infection*log(dose) + (1 | Study)	7	598.7	4.18	0.044
6	Mortality ~ T + mode of infection + life stage + (1 | Study)	7	599.2	4.75	0.033
7	Mortality ~ T + life stage + (1 | Study)	6	599.2	4.76	0.033
8	Mortality ~ T + log(dose) + life stage + (1 | Study)	7	600.9	6.42	0.014
9	Mortality ~ T + mode of infection + log(dose) + life stage + (1 | Study)	8	601.2	6.69	0.012
10	Mortality ~ T + mode of infection*log(dose) + life stage + (1 | Study)	9	603.7	9.25	0.003

Supplementary Table 7. Warm-water molluscs infected with *Vibrio*. Specific subset 7 (SSb 7).

Model ranking	Model	df	AICc	ΔAICc	w_i
Selection of random effects					
1	Mortality ~ T + (1 | pathogen species) + (1 | Study)	5	546.9	0.00	0.667
2	Mortality ~ T + (1 | pathogen species) + (1 | host family) + (1 | Study)	6	549.4	2.48	0.194
3	Mortality ~ T + (1 | Study)	4	550.6	3.66	0.107
4	Mortality ~ T + (1 | host family) + (1 | Study)	5	553.0	6.04	0.033
Selection of fixed effects (linear mixed model)					
1	Mortality ~ T + mode of infection*log(dose) + (1 | pathogen species) + (1 | Study)	7	548.6	0.00	0.431
**2**	**Mortality ~ T + log(dose) + (1 | pathogen species)** + (1 | Study)	**5**	**550.2**	**1.59**	**0.195**
3	Mortality ~ T + (1 | pathogen species) + (1 | Study)	4	550.6	2.00	0.159
4	Mortality ~ T + mode of infection + log(dose) + (1 | pathogen species) + (1 | Study)	6	552.6	3.99	0.059
5	Mortality ~ T + mode of infection + (1 | pathogen species) + (1 | Study)	5	552.8	4.27	0.051
6	Mortality ~ T + mode of infection*log(dose) + life stage + (1 | pathogen species) + (1 | Study)	9	553.2	4.58	0.044
7	Mortality ~ T + life stage + (1 | pathogen species) + (1 | Study)	6	554.3	5.69	0.025
8	Mortality ~ T + life stage + log(dose) + (1 | pathogen species) + (1 | Study)	7	554.3	5.77	0.024
9	Mortality ~ T + life stage + mode of infection (1 | pathogen species) + (1 | Study)	7	556.7	8.11	0.007
10	Mortality ~ T + mode of infection + log(dose) + life stage + (1 | pathogen species) + (1 | Study)	8	557.0	8.43	0.006

Supplementary Table 8. Warm-water (tropical and subtropical) host species infected by bacteria. General subset 1 (GSb 1). This model was constructed combining the datasets of infections caused by bacterial species in tropical and subtropical hosts that were significantly associated with temperature (*Aeromonas, Edwardsiella,* Streptococcae*, Vibrio*).

Model ranking	Model	df	AICc	ΔAICc	w_i
Selection of random effects					
1	Mortality ~ T + (1 | reference)	4	3019.7	0.00	0.234
2	Mortality ~ T + (1 | host family) + (1 | reference)	5	3020.2	0.49	0.183
3	Mortality ~ T + (1 | pathogen species) + (1 | host family) + (1 | reference)	6	3021.0	1.37	0.118
4	Mortality ~ T + (1 | pathogen species) + (1 | reference)	5	3021.4	1.70	0.100
5	Mortality ~ T + (1 | host phylum) + (1 | reference)	5	3021.6	1.91	0.090
6	Mortality ~ T + (1 | pathogen family) + (1 | reference)	5	3021.7	2.06	0.084
7	Mortality ~ T + (1 | host phylum/host family) + (1 | reference)	6	3022.0	2.38	0.071
8	Mortality ~ T + (1 | pathogen family/pathogen species) + (1 | host family) + (1 | reference)	7	3023.1	3.42	0.042
9	Mortality ~ T + (1 | host phylum) + (1 | pathogen species) + (1 | reference)	6	3023.2	3.51	0.041
10	Mortality ~ T + (1 | pathogen family/pathogen species) + (1 | reference)	6	3023.4	3.77	0.036
Selection of fixed effects (linear mixed model)					
**1**	**Mortality ~ T + mode of infection*log(dose) + (1 | Reference)**	**7**	**3015.7**	**0.00**	**0.575**
2	Mortality ~ T + mode of infection + (1 | Reference)	5	3018.8	3.17	0.118
3	Mortality ~ T + mode of infection*log(dose) + (1 | Reference)	10	3019.3	3.68	0.091
4	Mortality ~ T + (1 | Reference)	4	3019.7	4.01	0.078
5	Mortality ~ T + mode of infection + log(dose) + (1 | Reference)	6	3020.9	5.24	0.042
6	Mortality ~ T + life stage + mode of infection + (1 | Reference)	8	3021.0	5.34	0.040
7	Mortality ~ T + log(dose) + (1 | Reference)	5	3021.7	6.05	0.028
8	Mortality ~ T + life stage + mode of infection + log(dose) + (1 | Reference)	9	3023.1	7.39	0.014
9	Mortality ~ T + host life stage + (1 | Reference)	7	3023.7	8.06	0.010
10	Mortality ~ T + life stage + log(dose) + (1 | Reference)	8	3025.8	10.15	0.004

Supplementary Table 9. Temperate fish infected with *Aeromonas*. Specific subset 8 (SS8). Study was initially considered as a random effect, but it was detected as a singular variable (variance of random effect of 0), and therefore it was deleted to avoid overfitted models.

Model ranking	Model	df	AICc	ΔAICc	w_i
	Selection of random effects				
1	Mortality ~ T	3	577.5	0.00	0.577
2	Mortality ~ T + (1 | host family)	4	579.8	2.29	0.183
3	Mortality ~ T + (1 | pathogen species)	4	579.8	2.29	0.183
	Mortality ~ T + (1 | host family) + (1 | pathogen species)	5	582.1	4.67	0.056
	Selection of fixed effects (linear regression model)				
1	Mortality ~ T + mode of infection	4	577.4	0.00	0.208
**2**	**Mortality ~ T**	**5**	**577.5**	**0.09**	**0.199**
3	Mortality ~ T + log(dose)	3	577.7	0.36	0.174
4	Mortality ~ T + mode of infection + log(dose)	4	578.4	1.04	0.124
5	Mortality ~ T + mode of infection + life stage	5	579.6	2.28	0.067
6	Mortality ~ T + life stage	6	579.7	2.38	0.063
7	Mortality ~ T + life stage + log(dose)	6	580.0	2.65	0.055
8	Mortality ~ T + mode of infection*log(dose)	4	580.2	2.81	0.051
9	Mortality ~ T + life stage + mode of infection + log(dose)	5	580.6	3.26	0.041
10	Mortality ~ T + life stage + mode of infection* log(dose)	7	582.3	4.92	0.018

Supplementary Table 10. Temperate fish infected with *Edwardsiella*. Specific subset 9 (SSb 9). Study was initially considered as a random effect, but it was detected as a singular variable (variance of random effect of 0), and therefore it was deleted to avoid overfitted models.

Model ranking	Model	df	AICc	ΔAICc	w_i
Selection of random effects					
1	Mortality ~ T	3	159.6	0.00	0.729
2	Mortality ~ T + (1 | host family)	4	166.5	3.49	0.127
3	Mortality ~ T + (1 | pathogen species)	4	166.6	3.49	0.127
4	Mortality ~ T + (1 | pathogen species) + (1 | host family)	5	171.0	7.61	0.016
Selection of fixed effects (linear regression model)					
**1**	**Mortality ~ T + mode of infection**	**4**	**154.7**	**0.00**	**0.514**
2	Mortality ~ T + mode of infection*log(dose)	6	157.0	2.28	0.164
3	Mortality ~ T + mode of infection + log(dose)	5	158.0	3.31	0.098
4	Mortality ~ T + mode of infection + life stage	5	158.4	3.73	0.080
5	Mortality ~ T + log(dose)	4	159.2	4.54	0.053
6	Mortality ~ T	3	159.5	4.89	0.045
7	Mortality ~ T + life stage + mode of infection*log(dose)	7	161.7	7.03	0.015
8	Mortality ~ T + mode of infection + log(dose) + life stage	6	162.4	7.68	0.011
9	Mortality ~ T + life stage	4	162.4	7.69	0.011
10	Mortality ~ T + life stage + log(dose)	5	162.8	8.12	0.009

Supplementary Table 11. Temperate fish (salmonidae) infected with *Flavobacterium columnare*. Specific subset 10 (SS10). There was no need of evaluating random effects since only one pathogen species and one host family was present in the subset. Life stage and mode of infection were neither considered because all study points consisted in juvenile fish experimentally immersed in a bacterial solution to cause the disease.

Model ranking	Model	df	AICc	ΔAICc	w_i
Selection of fixed effects (linear regression model)					
**1**	**Mortality ~ T + log(dose)** + (1 | Study)	**5**	**410.0**	**0.00**	**0.59**
2	Mortality ~ T + (1 | Study)	4	410.8	0.73	0.41

Supplementary Table 12. Temperate shellfish infected with *Vibrio*. Specific subset 12 (SS12).

Model ranking	Model	df	AICc	ΔAICc	w_i
Selection of random effects					
1	Mortality ~ T + (1 | Study)	4	226.4	0.00	0.690
2	Mortality ~ T + (1 | pathogen species) + (1 | Study)	5	229.4	3.08	0.148
3	Mortality ~ T + (1 | host family) + (1 | Study)	5	229.6	3.23	0.137
4	Mortality ~ T + (1 | host family) + (1 | pathogen species) + (1 | Study)	6	233.0	6.68	0.024
Selection of fixed effects (linear regression model)					
**1**	**Mortality ~ T + mode of infection** + (1 | Study)	**5**	**223.9**	**0.00**	**0.503**
2	Mortality ~ T + (1 | Study)	4	226.4	2.49	0.145
3	Mortality ~ T + mode of infection + life stage + (1 | Study)	6	226.7	2.87	0.120
4	Mortality ~ T + mode of infection + log(dose) + (1 | Study)	6	227.1	3.19	0.102
5	Mortality ~ T + mode of infection*log(dose) + (1 | Study)	7	228.8	4.95	0.042
6	Mortality ~ T + log(dose) + (1 | Study)	5	229.5	5.61	0.030
7	Mortality ~ T + host life stage + (1 | Study)	5	229.5	5.61	0.030
8	Mortality ~ T + mode of infection + log(dose) + life stage + (1 | Study)	7	230.6	6.76	0.017
9	Mortality ~ T + log(dose) + life stage + (1 | Study)	6	232.8	8.90	0.006
10	Mortality ~ T + mode of infection*log(dose) + life stage + (1 | Study)	8	233.4	9.54	0.004

Supplementary Table 13. Temperate fish (Salmonidae) infected with *Yersinia ruckeri*. Specific subset 12 (SSb 12). The only random effect considered was study, since all studies concerned fish from salmonidae family and one pathogen species.

Model ranking	Model	df	AICc	ΔAICc	w_i
Selection of fixed effects (linear regression model)					
**1**	**Mortality ~ T + log(dose) + (1 | Study)**	**5**	**429.1**	**0.00**	**0.351**
2	Mortality ~ T + mode of infection + (1 | Study)	5	430.8	1.63	0.156
3	Mortality ~ T + mode of infection + log(dose) + (1 | Study)	6	431.3	2.21	0.116
4	Mortality ~ T + (1 | Study)	4	431.6	2.50	0.101
5	Mortality ~ T + life stage + log(dose) + (1 | Study)	6	431.7	2.56	0.098
6	Mortality ~ T + life stage + (1 | Study)	5	432.8	3.71	0.055
7	Mortality ~ T + mode of infection + life stage + (1 | Study)	6	433.0	3.88	0.050
8	Mortality ~ T + mode of infection*log(dose) + (1 | Study)	7	433.7	4.59	0.035
9	Mortality ~ T + mode of infection + log(dose) + life stage + (1 | Study)	7	434.1	4.93	0.030
10	Mortality ~ T + mode of infection*log(dose) + life stage + (1 | Study)	8	436.6	7.50	0.008

Supplementary Table 14. Temperate host species infected by bacteria. General subset 2 (GSb 2). This model was constructed combining the datasets of infections caused by bacterial species in temperate hosts that were significantly associated with temperature (*Aeromonas, Vibrio* and *F. columnare*). We initially included study as a random effect, however it led to singular fit models, when combined with other random effects (e.g. pathogen species). Therefore, the variable study was deleted, to avoid overfitted models.

Model ranking	Model	df	AICc	ΔAICc	w_i
Selection of random effects					
1	Mortality ~ T + (1 | pathogen species)	4	1214.3	0.00	0.356
2	Mortality ~ T + (1 | pathogen family)	4	1215.0	0.72	0.248
3	Mortality ~ T + (1 | pathogen species)+ (1 | host family)	5	1216.5	2.17	0.120
4	Mortality ~ T + (1 | pathogen species) + (1 | host phylum)	5	1216.5	2.17	0.120
5	Mortality ~ T + (1 | pathogen family/pathogen species)	5	1216.5	2.23	0.117
6	Mortality ~ T + (1 | pathogen family/pathogen species) + (1 | host family)	6	1218.7	4.43	0.039
7	Mortality ~ T + (1 | host family)	4	1233.2	18.94	0.000
8	Mortality ~ T	3	1233.9	19.59	0.000
9	Mortality ~ T + (1 | host phylum/host family)	5	1235.4	21.10	0.000
10	Mortality ~ T + (1 | host phylum)	4	1236.0	21.72	0.000
Selection of fixed effects (linear mixed model)					
**1**	**Mortality ~ T + mode of infection + (1 | pathogen species)**	**5**	**1209.7**	**0.00**	**0.511**
2	Mortality ~ T + mode of infection+ log(dose) + (1 | pathogen species)	6	1211.6	1.90	0.197
3	Mortality ~ T + mode of infection*log(dose)+ (1 | pathogen species)	7	1213.4	3.62	0.083
4	Mortality ~ T + mode of infection + life stage + (1 | pathogen species)	7	1213.4	3.64	0.083
5	Mortality ~ T + (1 | pathogen species)	4	1214.3	4.59	0.052
6	Mortality ~ T + mode of infection + log(dose) + life stage + (1 | pathogen species)	8	1215.4	5.72	0.029
7	Mortality ~ T + log(dose) + (1 | pathogen species)	5	1215.9	6.16	0.023
8	Mortality ~ T + mode of infection*log(dose) + life stage + (1 | pathogen species)	9	1217.2	7.47	0.012
9	Mortality ~ T + life stage + (1 | pathogen species)	6	1218.5	8.73	0.006
10	Mortality ~ T life stage + log(dose) + (1 | pathogen species)	7	1220.3	10.53	0.003

Supplementary Table 15. Details of the selected models for the different data subsets. P-value are not give for mixed-effect models, since they are considered statistically relevant in these models. In these models a parameter is considered significant when the 95% confidence intervals does not include 0.

Data subset	Model selected	Adj. R^2	Parameter	Estimate	SE	95% CI Lower	95% CI Upper
Aeromonas warm species	Mortality ~ T + (1 | Study)	0.529	T	2.68	0.74	1.15	4.28
Edwardsiella warm species	Mortality ~ T + Mode of infection*log(dose) + (1 | host family) + (1 | Study)	0.615	T	3.52	1.06	1.22	6.19
			Mode of infection	-25.22	22.33	-72.63	21.90
			Log(dose)	-2.38	0.97	-4.54	-0.41
			Mode of infection * log(dose)	3.47	1.57	0.29	6.75
F. columnare warm species	Mortality ~ T + log(dose) + (1 | Study)	0.080	T	2.69	1.91	-1.12	6.68
			Log(dose)	1.81	1.03	-0.44	3.86
Streptoccocae warm species	Mortality ~ T + mode of infection + (1 | host Study)	0.658	T	3.32	0.68	1.95	4.68
			Mode of infection	37.52	10.25	16.42	59.53
Vibrio warm arthropods	Mortality ~ T + mode of infection + (1 | Study)	0.649	T	3.77	0.98	1.82	5.78
			Mode of infection	-13.47	7.34	-28.10	1.39
Vibrio warm fish	Mortality ~ T + mode of infection	0.325	T	5.49	1.27	2.88	8.09
			Mode of infection	-19.11	10.28	-40.12	1.91
Vibrio warm molluscs	Mortality ~ T + mode of infection*log(dose) (1 | pathogen species) + (1 | Study)	0.774	T	4.54	0.55	3.37	5.64
			Mode of infection	-143.52	53.71	- 250.74	-35.45
			log(dose)	-5.62	3.69	-13.02	1.91
			Mode of infection*log(dose)	10.53	3.97	2.57	18.46
Aeromonas temperate	Mortality ~ T	0.13	T	3.80	1.19	1.41	6.19
Edwardsiella temperate	Mortality ~ T + Mode of infection	0.282	T	0.63	0.81	-1.09	2.36
			Mode of infection	19.14	6.54	5.28	33.01
F. columnare temperate	Mortality ~ T + log(dose) + (1 | Study)	0.736	T	5.60	0.63	4.21	6.90
			Log(dose)	11.31	5.36	-1.22	22.73
Vibrio temperate molluscs	Mortality ~ T + mode of infection	0.581	T	6.51	1.33	3.73	9.43
			Mode of infection	42.15	15.77	7.99	77.95
Y. *ruckerii* temperate	Mortality ~ T + log(dose) + (1 | Study)	0.321	T	2.19	1.82	-1.49	6.07
			Log(dose)	-2.10	0.91	-3.92	-0.27

Supplementary Table 16. Nested multiple regression models of aquaculture-related MAR and temperature, gdp per capita, clinical MAR and their interaction terms.

Model ranking	Model	*F*	Adj. R^2	P-value
1	MAR aquaculture ~ T + gdp + clinical MAR + T*gdp	3.757	0.29	0.017
2	MAR aquaculture ~ T + gdp + clinical MAR + T*clinical MAR*gdp	2.216	0.24	0.077
3	MAR aquaculture ~ T + gdp + clinical MAR + clinical MAR*gdp	2.812	0.21	0.049
4	MAR aquaculture ~ T + gdp + clinical MAR	3.276	0.20	0.038
5	MAR aquaculture ~ T + gdp + clinical MAR + T*clinical MAR	2.673	0.20	0.057

Supplementary Table 17. Antimicrobial intrinsic resistance of bacteria at genus level recovered from literature.

Bacterial genus	Intrinsic resistances	Reference
Aeromonas	Ampicillin	Hernould, M., Gagné, S., Fournier, M., Quentin, C. & Arpin, C. Role of the AheABC efflux pump in *Aeromonas hydrophila* intrinsic multidrug resistance. *Antimicrob. Agents Chemother.* **52**, 1559-1563 (2008).
Citrobacter	Ampicillin	Arens, S., Verhaegen, J. & Verbist, L. Differentiation and susceptibility of *Citrobacter* isolates from patiens in a university hospital. *Clin. Microbiol. Infect.* **3**, 53-57 (1997).
Edwardsiella	Cationic peptides (colistin, polymyxin B), lincosamides, oxacillin, streptogrammin	Stock. I. & Wiedemann, B. Natural antibiotic susceptibilities of *Edwardsiella tarda, E. ictaluri* and *E. hoshinae. Antimicrob. Agents Chemother.* **45**, 2245-2255 (2001). Santander, J., Martin, T., Loh, A., Pohlenz, C., Gatlin, D.M. & Curtis, R. Mechanisms of intrinsic resistance to antimicrobial peptides of *Edwardsiella ictaluri* and its influence on fish gut inflammation and virulence. *Microbiology* **159**, 1471-1486 (2013).
Enterobacter	Ampicillin, amoxicillin, clavunalate, first generation cephalosporins, cefoxitin	Daving-Regli, A. & Pagès, J-M. *Enterobacter aerogenes* and *Enterobacter cloacae;* versatile bacterial pathogens confronting antibiotic treatment. *Front. Microbiol.* **6,** 392 (2015).
Flavobacterium	Potentiated sulphonamides	Bruun, M.S., Schmidt, A.S., Madsen, L. & Dalsgaard, I. Antimicrobial resistance patterns in Danish isolates of *Flavobacterium psychrophilum. Aquaculture* **187**, 201-212 (2000).
Lactococcus	Clindamycin and vancomycin	Devirgilis, C., Zinno, P. & Perozzi, G. Update on antibiotic resistance in foodborne *Lactobacillus* and *Lactococcus* species. *Front. Microbiol.* **4,** 301 (2013).
Streptococcus	Cationic peptides	LaRock, C. & Nizet, V. Cationic antimicrobial peptide resistance mechanisms of Streptococcal pathogens. *Biochem. Biophys. Acta* **1848**, 3047-3054 (2015).
Vibrio	Ampicillin, penicillin	Chiou, J., Li, R., Chen, S. CARB-17 family of β-lactamases mediates intrinsic resistance to penicillins of *Vibrio parahaemolyticus. Antimicrob. Agents Chemother.* **59,** 3593-3595 (2015).
Yersinia	Benzylpenicillin, lincosamides, oxacillin, rifampicin	Stock, I., Henrichfreise, B., Wiedemann, B. Natural antibiotic susceptibility and biochemical profiles of *Yersinia enterolitica*-like strains: *Y. bercovieri, Y. mollaretti, Y. aldovae* and *Y. ruckeri. J. Med. Microbiol.* **51**, 56-69 (2002).
