# Supplementary material for: Aquaculture at the crossroads of global warming and antimicrobial resistance
Source: Nat Commun. 2020 Apr 20;11:1870. doi: 10.1038/s41467-020-15735-6 (PMC7170852; doi:10.1038/s41467-020-15735-6)
Supplement: Supplementary file 3 — Description of Additional Supplementary Information [file 41467_2020_15735_MOESM3_ESM.pdf]

Description of Additional Supplementary Files

File Name: Supplementary Data 1
Description: References used in the dataset that investigated the influence of temperature on the mortality of aquatic reared animals infected with virus or bacteria.

File Name: Supplementary Data 2
Description: References used in the dataset that investigated the antimicrobial resistance from aquaculture-related bacteria and that was used to calculate the Multi Antibiotic Index (MAR) for 40 countries.
