# Supplementary material for: Aquaculture at the crossroads of global warming and antimicrobial resistance
Source: Nat Commun. 2020 Apr 20;11:1870. doi: 10.1038/s41467-020-15735-6 (PMC7170852; doi:10.1038/s41467-020-15735-6)
Supplement: Supplementary file 4 — Supplementary Data 1 [file 41467_2020_15735_MOESM4_ESM.pdf]

Supplementary data 1: References used in the dataset that investigated the influence of temperature on the mortality of aquatic reared animals infected with virus or bacteria.

1. Abbass, A., Sharifuzzaman, S. M. & Austin, B. Cellular components of probiotics control *Yersinia ruckeri* infection in rainbow trout, *Oncorhynchus mykiss* (Walbaum). *J. Fish Dis.* **33**, 31–37 (2010).
2. Abdel‐Tawwab, M. & Ahmad, M. H. Live Spirulina (*Arthrospira platensis*) as a growth and immunity promoter for Nile tilapia, *Oreochromis niloticus* (L.), challenged with pathogenic *Aeromonas hydrophila*. *Aquac. Res.* **40**, 1037–1046 (2009).
3. Abutbul, S., Golan-Goldhirsh, A., Barazani, O. & Zilberg, D. Use of *Rosmarinus officinalis* as a treatment against *Streptococcus iniae* in tilapia (*Oreochromis sp.*). *Aquaculture* **238**, 97–105 (2004).
4. Acar, Ü., Kesbiç, O. S., Yılmaz, S., Gültepe, N. & Türker, A. Evaluation of the effects of essential oil extracted from sweet orange peel (*Citrus sinensis*) on growth rate of tilapia (*Oreochromis mossambicus*) and possible disease resistance against *Streptococcus iniae*. *Aquaculture* **437**, 282–286 (2015).
5. Acar, Ü. *et al.* Effects of different levels of pomegranate seed oil on some blood parameters and disease resistance against *Yersinia ruckeri* in rainbow trout. *Front. Physiol.* **9**, (2018).
6. Adams, A., Leschen, W., Wilson, A. & Horne, M. T. A bath challenge model for furunculosis in rainbow trout*, Salmo gairdneri* Richardson, and Atlantic salmon, *Salmo salar* L. *J. Fish Dis.* **10**, 495–504 (1987).
7. Adel, M., Pourgholam, R., Zorriehzahra, J. & Ghiasi, M. Hemato – Immunological and biochemical parameters, skin antibacterial activity, and survival in rainbow trout (*Oncorhynchus mykiss*) following the diet supplemented with *Mentha piperita* against *Yersinia ruckeri*. *Fish Shellfish Immunol.* **55**, 267–273 (2016).
8. Akhlaghi, M. & Sharifi Yazdi, H. Detection and identification of virulent *Yersinia ruckeri*: the causative agent of enteric redmouth disease in rainbow trout (*Oncorhynchus mykiss*) cultured in Fars province, Iran. *Iranian J. Vet. Res.* **9**, 347–352 (2008).
9. Alavandi, S. V., Manoranjita, V., Vijayan, K. K., Kalaimani, N. & Santiago, T. C. Phenotypic and molecular typing of *Vibrio harveyi* isolates and their pathogenicity to tiger shrimp larvae. *Lett. Appl. Microbiol.* **43**, 566–570 (2006).
10. Alcaide, E., Amaro, C., Todolí, R. & Oltra, R. Isolation and characterization of *Vibrio parahaemolyticus* causing infection in Iberian toothcarp *Aphanius iberus*. *Dis. Aquat. Org.* **35**, 77–80 (1999).
11. Al-Faragi, J. K. & Hassan, M. A. H. Efficiency of dietary turmeric on growth performance, hematology and survival rate in common carp *Cyprinus carpio* challenged with *Flexibacter columnaris*. *Kufa J. Vet. Med. Sci.* **8**, (2017).
12. Allam, B., Paillard, C. & Ford, S. E. Pathogenicity of *Vibrio tapetis*, the etiological agent of brown ring disease in clams. *Dis. Aquat. Org.* **48**, 221–231 (2002).
13. Altinok, I. & Grizzle, J. M. Effects of salinity on *Yersinia ruckeri* infection of rainbow trout and brown trout. *J. Aquat. Anim. Health* **13**, 334–339 (2001).
14. Altinok. The infectious route of *Yersinia ruckeri* is affected by salinity. *Bull. Eur. Ass. Fish Pathol.* **24**, 253-259 (2004).
15. Amphan, S., Unajak, S., Printrakoon, C. & Areechon, N. Feeding-regimen of β-glucan to enhance innate immunity and disease resistance of Nile tilapia, *Oreochromis niloticus* Linn., against *Aeromonas hydrophila* and *Flavobacterium columnare*. *Fish Shellfish Immunol.* **87**, 120–128 (2019).
16. Anderson, D. P. & Siwicki, A. K. Duration of protection against *Aeromonas salmonicida* in brook trout Immunostimulated with glucan or chitosan by injection or immersion. *Progressive Fish-Culturist* **56**, 258–261 (1994).
17. Anshary, H., Kurniawan, R. A., Sriwulan, S., Ramli, R. & Baxa, D. V. Isolation and molecular

identification of the etiological agents of streptococcosis in Nile tilapia (*Oreochromis niloticus*) cultured in net cages in Lake Sentani, Papua, Indonesia. *SpringerPlus* **3**, 627 (2014).
18. Arias, C. R., Cai, W., Peatman, E. & Bullard, S. A. Catfish hybrid *Ictalurus punctatus* × *I. furcatus* exhibits higher resistance to columnaris disease than the parental species. *Dis. Aquat. Org.* **100**, 77–81 (2012).
19. Austin, D. A., Robertson, P. A. W. & Austin, B. Recovery of a new biogroup of *Yersinia ruckeri* from diseased rainbow trout (*Oncorhynchus mykiss*, Walbaum). *Syst. App. Microbiol.* **26**, 127–131 (2003).
20. Azeredo, R. *et al.* European sea bass (*Dicentrarchus labrax*) immune status and disease resistance are impaired by arginine dietary supplementation. *PLoS ONE* **10**, e0139967 (2015).
21. Baba, E., Acar, Ü., Öntaş, C., Kesbiç, O. S. & Yilmaz, S. The use of *Avena sativa* extract against *Aeromonas hydrophila* and its effect on growth performance, hematological and immunological parameters in common carp (*Cyprinus carpio*). *Italian J Anim. Sci.* **15**, 325–333 (2016).
22. Baba, E., Acar, Ü., Öntaş, C., Kesbiç, O. S. & Yılmaz, S. Evaluation of *Citrus limon* peels essential oil on growth performance, immune response of Mozambique tilapia *Oreochromis mossambicus* challenged with *Edwardsiella tarda*. *Aquaculture* **465**, 13–18 (2016).
23. Baba, E., Acar, Ü., Yılmaz, S., Öntaş, C. & Kesbiç, O. S. Pre-challenge and post-challenge haemato-immunological changes in *Oreochromis niloticus* (Linnaeus, 1758) fed argan oil against *Lactococcus garvieae*. *Aquac. Res.* **48**, 4563–4572 (2017).
24. Baba, E., Acar, Ü., Yılmaz, S., Zemheri, F. & Ergün, S. Dietary olive leaf (*Olea europea* L.) extract alters some immune gene expression levels and disease resistance to *Yersinia ruckeri* infection in rainbow trout *Oncorhynchus mykiss*. *Fish Shellfish Immunol.* **79**, 28–33 (2018).
25. Bader, J. A., Nusbaum, K. E. & Shoemaker, C. A. Comparative challenge model of *Flavobacterium columnare* using abraded and unabraded channel catfish, *Ictalurus punctatus* (Rafinesque). *J. Fish Dis.* **26**, 461–467 (2003).
26. Balcázar, J. L., Rojas-Luna, T. & Cunningham, D. P. Effect of the addition of four potential probiotic strains on the survival of pacific white shrimp (*Litopenaeus vannamei*) following immersion challenge with *Vibrio parahaemolyticus*. *J. Invertebr. Pathol.* **96**, 147–150 (2007).
27. Balebona, M. C. *et al.* Pathogenicity of *Vibrio alginolyticus* for cultured gilt-head sea bream (*Sparus aurata* L.). *Appl. Environ. Microbiol.* **64**, 4269–4275 (1998).
28. Basha, K. A. *et al.* Effect of dietary supplemented andrographolide on growth, non-specific immune parameters and resistance against *Aeromonas hydrophila* in *Labeo rohita* (Hamilton). *Fish Shellfish Immunol.* **35**, 1433–1441 (2013).
29. Bastardo, A., Ravelo, C. & Romalde, J. L. Highly sensitive detection and quantification of the pathogen *Yersinia ruckeri* in fish tissues by using real-time PCR. *Appl. Microbiol. Biotechnol.* **96**, 511–520 (2012).
30. Baxa, D. V., Groff, J. M., Wishkovsky, A. & Hedrick, R. P. Susceptibility of nonictalurid fishes to experimental infection with *Edwardsiella ictaluri*. *Dis. Aquat. Org.* **8**, 113–117 (1990).
31. Beck, B. H., Barnett, L. M., Farmer, B. D., Peatman, E. & Carter, D. Kaolinitic clay protects against *Flavobacterium columnare* infection in channel catfish *Ictalurus punctatus* (Rafinesque). *J. Fish Dis.* **38**, 241–248 (2015).
32. Biering, E., Vaagnes, Ø., Krossøy, B., Gulla, S. & Colquhoun, D. J. Challenge models for atypical *Aeromonas salmonicida* and *Vibrio anguillarum* in farmed Ballan wrasse (*Labrus bergylta*) and preliminary testing of a trial vaccine against atypical *Aeromonas salmonicida*. *J. Fish Dis.* **39**, 1257–1261 (2016).

33. Bilodeau-Bourgeois, L., Bosworth, B. G. & Peterson, B. C. Differences in mortality, growth, lysozyme, and Toll-like receptor gene expression among genetic groups of catfish exposed to virulent *Edwardsiella ictaluri*. *Fish Shellfish Immunol.* **24**, 82–89 (2008).
34. Björnsdóttir, B., Gudmundsdóttir, S., Bambir, S. H. & Gudmundsdóttir, B. K. Experimental infection of turbot, *Scophthalmus maximus* (L.), by *Aeromonas salmonicida subsp. achromogenes* and evaluation of cross protection induced by a furunculosis vaccine. *J. Fish Dis.* **28**, 181–188 (2005).
35. Blanch, A. R., Pintó, R. M. & Jofre, J. T. Isolation and characterization of an *Edwardsiella sp*. strain, causative agent of mortalities in sea bass (*Dicentrarchus labrax*). *Aquaculture* **88**, 213–222 (1990).
36. Bowser, P. R., Wooster, G. A. & Hsu, H.-M. Laboratory efficacy of enrofloxacin for the control of *Aeromonas salmonicida* infection in rainbow trout. *J. Aquat. Anim. Health* **6**, 288–291 (1994).
37. Breyer, K. E. *et al.* Efficacy of an extract from garlic, *Allium sativum*, against infection with the furunculosis bacterium, *Aeromonas salmonicida*, in rainbow trout, *Oncorhynchus mykiss*. *J. World Aquac. Soc.* **46**, 273–282 (2015).
38. Bricknell, I. R. *et al.* Susceptibility of Atlantic halibut, *Hippoglossus hippoglossus* (L.) to infection with typical and atypical *Aeromonas salmonicida*. *Aquaculture* **175**, 1–13 (1999).
39. Bromage, E. & Owens, L. Environmental factors affecting the susceptibility of barramundi to *Streptococcus iniae*. *Aquaculture* **290**, 224–228 (2009).
40. Buentello, J. A. & Gatlin, D. M. Effects of elevated dietary arginine on resistance of channel catfish to exposure to *Edwardsiella ictaluri. J. Aquat. Anim. Health* **13**, 194–201 (2001).
41. Cai, J., Han, Y. & Wang, Z. Isolation of *Vibrio parahaemolyticus* from abalone (*Haliotis diversicolor supertexta* L.) postlarvae associated with mass mortalities. *Aquaculture* **257**, 161–166 (2006).
42. Cai, J., Li, J., Thompson, K. D., Li, C. & Han, H. Isolation and characterization of pathogenic *Vibrio parahaemolyticus* from diseased post-larvae of abalone *Haliotis diversicolor supertexta*. *J. Basic Microbiol.* **47**, 84–86 (2007).
43. Capkin, E. & Altinok, I. Effects of dietary probiotic supplementations on prevention/treatment of yersiniosis disease. *J. Appl. Microbiol.* **106**, 1147–1153 (2009).
44. Cardinaud, M., Dheilly, N. M., Huchette, S., Moraga, D. & Paillard, C. The early stages of the immune response of the European abalone *Haliotis tuberculata* to a *Vibrio harveyi* infection. *Dev. Comp. Immunol.* **51**, 287–297 (2015).
45. Castex, M., Lemaire, P., Wabete, N. & Chim, L. Effect of probiotic *Pediococcus acidilactici* on antioxidant defences and oxidative stress of *Litopenaeus stylirostris* under *Vibrio nigripulchritudo* challenge. *Fish Shellfish Immunol.* **28**, 622–631 (2010).
46. Chang, Y.-P. *et al.* Dietary administration of zingerone to enhance growth, non-specific immune response, and resistance to *Vibrio alginolyticus* in Pacific white shrimp (*Litopenaeus vannamei*) juveniles. *Fish Shellfish Immunol.* **32**, 284–290 (2012).
47. Chen, M. *et al.* PCR detection and PFGE genotype analyses of streptococcal clinical isolates from tilapia in China. *Vet. Microbiol.* **159**, 526–530 (2012).
48. Cheng, W., Hsiao, I.-S., Hsu, C.-H. & Chen, J.-C. Change in water temperature on the immune response of Taiwan abalone *Haliotis diversicolor supertexta* and its susceptibility to *Vibrio parahaemolyticus*. *Fish Shellfish Immunol.* **17**, 235–243 (2004).
49. Cheng, W., Juang, F.-M. & Chen, J.-C. The immune response of Taiwan abalone *Haliotis diversicolor supertexta* and its susceptibility to *Vibrio parahaemolyticus* at different salinity levels. *Fish Shellfish Immunol.* **16**, 295–306 (2004).
50. Cheng, W., Wang, L.-U. & Chen, J.-C. Effect of water temperature on the immune response of white shrimp *Litopenaeus vannamei* to *Vibrio alginolyticus*. *Aquaculture* **250**, 592–601 (2005).
51. Chideroli, R. T. *et al.* Emergence of a new multidrug-resistant and highly virulent serotype of

Streptococcus agalactiae in fish farms from Brazil. *Aquaculture* **479**, 45–51 (2017).
52. Cipriano, R. C. & Starliper, C. E. Immersion and injection vaccination of salmonids against furunculosis with an avirulent strain of *Aeromonas Salmonicida*. *Progressive Fish-Culturist* **44**, 167–169 (1982).
53. Crumlish, M., Thanh, P. C., Koesling, J., Tung, V. T. & Gravningen, K. Experimental challenge studies in Vietnamese catfish, *Pangasianodon hypophthalmus* (Sauvage), exposed to *Edwardsiella ictaluri* and *Aeromonas hydrophila*. *J. Fish Dis.* **33**, 717–722 (2010).
54. Darwish, A. M., Mitchell, A. & Straus, D. L. Evaluation of a 4-h static copper sulphate treatment against experimental infection of *Flavobacterium columnare* in channel catfish (*Ictalurus punctatus*). *Aquac. Res.* **43**, 688–695 (2012).
55. Dash, S. S. *et al.* Biochemical and serological characterization of *Flavobacterium columnare* from freshwater fishes of Eastern India. *J. World Aquac. Soc.* **40**, 236–247 (2009).
56. Dautremepuits, C., Fortier, M., Croisetiere, S., Belhumeur, P. & Fournier, M. Modulation of juvenile brook trout (*Salvelinus fontinalis*) cellular immune system after *Aeromonas salmonicida* challenge. *Vet. Immunol. Immunopathol.* **110**, 27–36 (2006).
57. Decostere, A., Haesebrouck, F. & Devriese, L. A. Characterization of four *Flavobacterium columnare* (*Flexibacter columnaris*) strains isolated from tropical fish. *Vet. Microbiol.* **62**, 35–45 (1998).
58. De Decker, S. *et al.* Responses of diploid and triploid Pacific oysters *Crassostrea gigas* to *Vibrio* infection in relation to their reproductive status. *J. Invertebr. Pathol.* **106**, 179–191 (2011).
59. de la Peña, L. D., Lavilla-Pitogo, C. R. & Paner, M. G. Luminescent vibrios associated with mortality in pond-cultured shrimp *Penaeus monodon* in the Philippines: species composition. *Fish Pathol.* **36**, 133–138 (2001).
60. Devi, T. B., Abraham, T. J. & Kamilya, D. Susceptibility and pathological consequences of catla, *Catla catla* (Hamilton) experimentally infected with *Edwardsiella tarda*. *Fisheries Aquat. Life* **24**, 209–217 (2016).
61. Dong, H. T. *et al.* Concurrent infections of *Flavobacterium columnare* and *Edwardsiella ictaluri* in striped catfish, *Pangasianodon hypophthalmus* in Thailand. *Aquaculture* **448**, 142–150 (2015).
62. Dong, H. T. *et al.* Natural occurrence of edwardsiellosis caused by *Edwardsiella ictaluri* in farmed hybrid red tilapia (*Oreochromis* sp.) in Southeast Asia. *Aquaculture* **499**, 17–23 (2019).
63. Dong, H. T., Senapin, S., LaFrentz, B. & Rodkhum, C. Virulence assay of rhizoid and non-rhizoid morphotypes of *Flavobacterium columnare* in red tilapia, *Oreochromis sp.,* fry. *J. Fish Dis.* **39**, 649–655 (2016).
64. Dong, H. T. *et al. Aeromonas jandaei* and *Aeromonas veronii* caused disease and mortality in Nile tilapia, *Oreochromis niloticus* (L.). *J. Fish Dis.* **40**, 1395–1403 (2017).
65. Dong, X. *et al.* An isolate of *Vibrio campbellii* carrying the pirVP gene causes acute hepatopancreatic necrosis disease. *Emerg. Microbes Infect.* **6**, e2 (2017).
66. Dubert, J., Romalde, J. L., Prado, S. & Barja, J. L. *Vibrio bivalvicida* sp. nov., a novel larval pathogen for bivalve molluscs reared in a hatchery. *Syst. Appl. Microbiol.* **39**, 8–13 (2016).
67. Dubief, B., Nunes, F. L. D., Basuyaux, O. & Paillard, C. Immune priming and portal of entry effectors improve response to *Vibrio* infection in a resistant population of the European abalone. *Fish Shellfish Immunol.* **60**, 255–264 (2017).
68. El-Asely, A. M., Abbass, A. A. & Austin, B. Honey bee pollen improves growth, immunity and protection of Nile tilapia (*Oreochromis niloticus*) against infection with *Aeromonas hydrophila*. *Fish Shellfish Immunol.* **40**, 500–506 (2014).
69. El-Boshy, M. E., El-Ashram, A. M., Abdelhamid, F. M. & Gadalla, H. A. Immunomodulatory

effect of dietary *Saccharomyces cerevisiae*, beta-glucan and laminaran in mercuric chloride treated Nile tilapia (*Oreochromis niloticus*) and experimentally infected with *Aeromonas hydrophila. Fish Shellfish Immunol.* **28**, 802–808 (2010).
70. El-Bouhy, Z. M., El-Nobi, G., Reda, R. M. & Ali, S. A. Prevalence of septicemia and red mouth disease caused by *Aeromonas sobria* at Sahl El-Housinia fish farm. *Zag. Vet. J.* **43**, (2016).
71. Evans, J. J., Klesius, P. H. & Shoemaker, C. A. First isolation and characterization of *Lactococcus garvieae* from Brazilian Nile tilapia, *Oreochromis niloticus* (L.), and pintado, *Pseudoplathystoma corruscans* (Spix & Agassiz). *J. Fish Dis.* **32**, 943–951 (2009).
72. Evenhuis, J. P., LaPatra, S. E. & Marancik, D. Early life stage rainbow trout (*Oncorhynchus mykiss*) mortalities due to *Flavobacterium columnare* in Idaho, USA. *Aquaculture* **418–419**, 126–131 (2014).
73. Evenhuis, J. P., Mohammed, H., LaPatra, S. E., Welch, T. J. & Arias, C. R. Virulence and molecular variation of *Flavobacterium columnare* affecting rainbow trout in Idaho, USA. *Aquaculture* **464**, 106–110 (2016).
74. Farmer, B. D., Beck, B. H., Mitchell, A. J., Rawles, S. D. & Straus, D. L. Dietary copper effects survival of channel catfish challenged with *Flavobacterium columnare*. *Aquac. Res.* **48**, 1751–1758 (2017).
75. Farto, R., Milton, D. L., Bermúdez, M. B. & Nieto, T. P. Colonization of turbot tissues by virulent and avirulent *Aeromonas salmonicida* subsp. *salmonicida* strains during infection. *Dis. Aquat. Org.* **95**, 167–173 (2011).
76. Fernández-Álvarez, C., Gijón, D., Álvarez, M. & Santos, Y. First isolation of *Aeromonas salmonicida* sbsp. *salmonicida* from diseased sea bass, *Dicentrarchus labrax* (L.), cultured in Spain. *Aquac. Rep.* **4**, 36–41 (2016).
77. Figueiredo, H. C. P. *et al.* Isolation and characterization of strains of *Flavobacterium columnare* from Brazil. *J. Fish Dis.* **28**, 199–204 (2005).
78. Flores-Miranda, M. del C. *et al.* Microbial immunostimulants reduce mortality in whiteleg shrimp (*Litopenaeus vannamei*) challenged with *Vibrio sinaloensis* strains. *Aquaculture* **320**, 51–55 (2011).
79. Fouz, B., Zarza, C. & Amaro, C. First description of non-motile *Yersinia ruckeri* serovar I strains causing disease in rainbow trout, *Oncorhynchus mykiss* (Walbaum), cultured in Spain. *J. Fish Dis.* **29**, 339–346 (2006).
80. Fu, S., Tian, H., Wei, D., Zhang, X. & Liu, Y. Delineating the origins of *Vibrio parahaemolyticus* isolated from outbreaks of acute hepatopancreatic necrosis disease in Asia by the use of whole genome sequencing. *Front. Microbiol.* **8**, (2017).
81. Galindo-Villegas, J., Ehime U., Fukada, H., Masumoto, T. & Hosokawa, H. Effect of dietary immunostimulants on some innate immune responses and disease resistance against *Edwardsiella tarda* infection in Japanese flounder (*Paralichthys olivaceus*). *Aquac. Sci. (Japan)* (2006).
82. Garcia, F., Moraes, F. R. & Martins, M. L. Challenge of pacu (*Piaractus mesopotamicus*) fed diets supplemented with vitamins C and E by *Aeromonas hydrophila* under different temperature. *Arq. Bras. Med. Vet. Zootec.* **61**, 378–385 (2009).
83. Gaunt, P. S., Chatakondi, N., Gao, D. & Endris, R. Efficacy of florfenicol for control of mortality associated with *Edwardsiella ictaluri* in three species of catfish. *J. Aquat. Anim. Health* **27**, 45–49 (2015).
84. Gay, M., Renault, T., Pons, A.-M. & Le Roux, F. Two *Vibrio splendidus* related strains collaborate to kill *Crassostrea gigas*: taxonomy and host alterations. *Dis. Aquat. Org.* **62**, 65–74 (2004).
85. Geng, Y. *et al. Streptococcus agalactiae*, an emerging pathogen for cultured Ya-Fish, *Schizothorax prenanti,* in China. *Trans. Emerg. Dis.* **59**, 369–375 (2012).
86. Geng, Y. *et al.* Isolation and characterization of *Edwardsiella ictaluri* from Southern catfish,

Silurus soldatovi meridionalis, (Chen) Cultured in China. *J. World Aquac. Soc.* **44**, 273–281 (2013).

87. Geng, Y., Wang, K., Chen, D., Fan, F., Huang, Y. Isolation and characterization of *Edwardsiella ictaluri* from cultured yellow catfish (*Pelteobagrus fulvidraco*). *Israeli J. Aquacult. Bamidgeh* **62**, 105-115 (2010)
88. Giri, S. S. *et al.* Effect of guava leaves on the growth performance and cytokine gene expression of *Labeo rohita* and its susceptibility to *Aeromonas hydrophila* infection. *Fish Shellfish Immunol.* **46**, 217–224 (2015).
89. Gómez-León, J., Villamil, L., Lemos, M. L., Novoa, B. & Figueras, A. Isolation of *Vibrio alginolyticus* and *Vibrio splendidus* from aquacultured carpet shell clam (*Ruditapes decussatus*) larvae associated with mass mortalities. *Appl. Environ. Microbiol.* **71**, 98–104 (2005).
90. Gravningen, K., Kestin, S., Thorarinsson, R. & Syvertsen, C. Oral vaccination against enteric red mouth disease in rainbow trout (*Oncorhynchus mykiss* Walbaum). The effect of vaccine dose rate on protection against the disease. *J. Appl. Ichthyol.* **14**, 163–166 (1998).
91. Griffin, B. R. & Mitchell, A. J. Susceptibility of channel catfish, *Ictalurus punctatus* (Rafinesque), to *Edwardsiella ictaluri* challenge following copper sulphate exposure. *J. Fish Dis.* **30**, 581–585 (2007).
92. Gültepe, N., Bilen, S., Yılmaz, S., Güroy, D. & Aydın, S. Effects of herbs and spice on health status of tilapia (*Oreochromis mossambicus*) challenged with *Streptococcus iniae*. *Acta Vet. Brno* **83**, 125–131 (2014).
93. Guz, L., Sopinska, A. & Oniszczuk, T. Effect of *Echinacea purpurea* on growth and survival of guppy (*Poecilia reticulata*) challenged with *Aeromonas bestiarum*. *Aquac. Nutr.* **17**, 695–700 (2011).
94. Haig, S. J., Davies, R. L., Welch, T. J., Reese, R. A. & Verner-Jeffreys, D. W. Comparative susceptibility of Atlantic salmon and rainbow trout to *Yersinia ruckeri*: Relationship to O antigen serotype and resistance to serum killing. *Vet. Microbiol.* **147**, 155–161 (2011).
95. Haldar, S. *et al.* Identification of *Vibrio harveyi* as a causative bacterium for a tail rot disease of sea bream *Sparus aurata* from research hatchery in Malta. *Microbiol. Res.* **165**, 639–648 (2010).
96. Han, H.-J., Kim, D.-H., Lee, D.-C., Kim, S.-M. & Park, S.-I. Pathogenicity of *Edwardsiella tarda* to olive flounder, *Paralichthys olivaceus* (Temminck & Schlegel). *J. Fish Dis.* **29**, 601–609 (2006).
97. Han, H.-J. *et al.* Atypical *Aeromonas salmonicida* infection in the black rockfish, *Sebastes schlegeli* Hilgendorf, in Korea. *J. Fish Di.s* **34**, 47–55 (2011).
98. Harikrishnan, R., Balasundaram, C. & Heo, M.-S. Influence of diet enriched with green tea on innate humoral and cellular immune response of kelp grouper (*Epinephelus bruneus*) to *Vibrio carchariae* infection. *Fish Shellfish Immunol.* **30**, 972–979 (2011).
99. Harikrishnan, R. *et al.* Phytotherapy of *Aeromonas hydrophila*-infected Goldfish, *Carassius auratus*. *J. World Aquac. Soc.* **41**, 391–401 (2010).
100. Harikrishnan, R. *et al.* Effect of *Agaricus bisporus* enriched diet on growth, hematology, and immune protection in *Clarias gariepinus* against *Flavobacterium columnare*. *Fish Shellfish Immunol.* **73**, 245–251 (2018).
101. Hashem, M. & El-Barbary, M. *Vibrio harveyi* infection in Arabian Surgeon fish (*Acanthurus sohal*) of Red Sea at Hurghada, Egypt. *The Egyptian Journal of Aquatic Research* **39**, 199–203 (2013).
102. Hassan, M. A., Noureldin, E. A., Mahmoud, M. A. & Fita, N. A. Molecular identification and epizootiology of Aeromonas veronii infection among farmed Oreochromis niloticus in Eastern Province, KSA. *Egypt. J. Aquat. Res.* **43**, 161–167 (2017).
103. Hedrick, R. P., Waltzek, T. B. & McDowell, T. S. Susceptibility of koi carp, common carp,

goldfish, and goldfish × common carp hybrids to cyprinid herpesvirus-2 and herpesvirus-3. *J. Aquat. Anim. Health* **18**, 26–34 (2006).
104. Hedrick, R. P. *et al.* A Herpesvirus associated with mass mortality of juvenile and adult koi, a strain of common carp. *J. Aquat. Anim. Health* **12**, 44–57 (2000).
105. Holt, R. A., Sanders, J. E., Zinn, J. L., Fryer, J. L. & Pilcher, K. S. Relation of water temperature to *Flexibacter columnaris* Infection in steelhead trout (*Salmo gairdneri*), Coho (*Oncorhynchus kisutch*) and Chinook (*O. tshawytscha*) Salmon. *J. Fish. Res. Bd. Can.* **32**, 1553–1559 (1975).
106. Hossain, M. F., Rahman, M. M. & Sayed, M. A. Experimental infection of indigenous climbing perch *Anabas testudineus* with *Aeromonas hydrophila* bacteria. *Progress. Agric.* **22**, 105–114 (2011).
107. Hossain, M. J. *et al.* An Asian origin of virulent *Aeromonas hydrophila* responsible for disease epidemics in United States-farmed catfish. *mBio* **5**, e00848-14 (2014).
108. Hsu, S.-W. & Chen, J.-C. The immune response of white shrimp *Penaeus vannamei* and its susceptibility to *Vibrio alginolyticus* under sulfide stress. *Aquaculture* **271**, 61–69 (2007).
109. Huang, C. Y., Liu, P. C. & Lee, K. K. Withering syndrome of the small abalone, *Haliotis diversicolor supertexta*, is caused by *Vibrio parahaemolyticus* and associated with thermal induction. *Z. Naturforsch., C, J. Biosci.* **56**, 898–901 (2001).
110. Ibrahem, M.D., Iman, S.B., El-Yazeed, A.H., Korani, H. Assessment of the susceptibility of polyculture reared African catfish and Nile tilapia to *Edwardsiella tarda*. *J. American Sci.* **7**, 779–786 (2011).
111. Inglis, V., Richards, R. H., Varma, K. J., Sutherland, I. H. & Brokken, E. S. Florfenicol in Atlantic salmon, *Salmo salar* L., parr: tolerance and assessment of efficacy against furunculosis. *J. Fish Dis.* **14**, 343–351 (1991).
112. Jayaprakash, N. S., Pai, S. S., Philip, R. & Singh, I. S. B. Isolation of a pathogenic strain of *Vibrio alginolyticus* from necrotic larvae of *Macrobrachium rosenbergii* (de Man). *J. Fish Dis.* **29**, 187–191 (2006).
113. Jeney, G., Ardó, L., Rónyai, A., Bercsényi, M. & Jeney, Z. Resistance of genetically different common carp, *Cyprinus carpio* L., families against experimental bacterial challenge with *Aeromonas hydrophila*. *J. Fish Dis.* **34**, 65–70 (2011).
114. Jiang, Q., Shi, L., Ke, C., You, W. & Zhao, J. Identification and characterization of *Vibrio harveyi* associated with diseased abalone Haliotis diversicolor. *Dis. Aquat. Org.* **103**, 133–139 (2013).
115. Karunasagar, I., Pai, R., Malathi, G. R. & Karunasagar, I. Mass mortality of *Penaeus monodon* larvae due to antibiotic-resistant *Vibrio harveyi* infection. *Aquaculture* **128**, 203–209 (1994).
116. Kayansamruaj, P., Pirarat, N., Katagiri, T., Hirono, I. & Rodkhum, C. Molecular characterization and virulence gene profiling of pathogenic *Streptococcus agalactiae* populations from tilapia (*Oreochromis sp.*) farms in Thailand. *J. Vet. Diagn. Invest.* **26**, 488–495 (2014).
117. Khouadja, S., Lamari, F. & Bakhrouf, A. Characterization of *Vibrio parahaemolyticus* isolated from farmed sea bass (*Dicentrarchus labrax*) during disease outbreaks. *Int. Aquat. Res.* **5**, 13 (2013).
118. Kiiyukia, C. *et al.* *Vibrio cholerae* non-O1 isolated from ayu fish (*Plecoglossus altivelis*) in Japan. *Appl. Environ. Microbiol.* **58**, 3078–3082 (1992).
119. Kitikiew, S. *et al.* Fucoidan effectively provokes the innate immunity of white shrimp *Litopenaeus vannamei* and its resistance against experimental *Vibrio alginolyticus* infection. *Fish Shellfish Immunol.* **34**, 280–290 (2013).
120. Kole, S. *et al.* Tissue specific expression profile of some immune related genes in *Labeo rohita* to *Edwardsiella tarda* infection. *Fish Shellfish Immunol.* **66**, 575–582 (2017).
121. Kongnum, K. & Hongpattarakere, T. Effect *of Lactobacillus plantarum* isolated from

digestive tract of wild shrimp on growth and survival of white shrimp (*Litopenaeus vannamei*) challenged with *Vibrio harveyi*. *Fish Shellfish Immunol.* **32**, 170–177 (2012).
122. Lacoste, A. *et al.* A *Vibrio splendidus* strain is associated with summer mortality of juvenile oysters *Crassostrea gigas* in the Bay of Morlaix (North Brittany, France). *Dis. Aquat. Org.* **46**, 139–145 (2001).
123. LaFrentz, B. R., LaPatra, S. E., Shoemaker, C. A. & Klesius, P. H. Reproducible challenge model to investigate the virulence of *Flavobacterium columnare* genomovars in rainbow trout *Oncorhynchus mykiss. Dis. Aquat. Org.* **101**, 115–122 (2012).
124. Lago, E. P., Nieto, T. P. & Farto, R. Virulence factors of *Aeromonas salmonicida* subsp. *salmonicida* strains associated with infections in turbot *Psetta maxima*. *Dis. Aquat. Org.* **99**, 145–151 (2012).
125. Lan, J., Zhang, X.-H., Wang, Y., Chen, J. & Han, Y. Isolation of an unusual strain of *Edwardsiella tarda* from turbot and establish a PCR detection technique with the gyrB gene. *J. Appl. Microbiol.* **105**, 644–651 (2008).
126. Lee, K.-K., Liu, P.-C., Chen, Y.-C. & Huang, C.-Y. The implication of ambient temperature with the outbreak of vibriosis in cultured small abalone *Haliotis diversicolor supertexta* Lischke. *J. Therm. Biol.* **26**, 585–587 (2001).
127. Lee, K. K., Yu, S. R., Yang, T. I., Liu, P. C. & Chen, F. R. Isolation and characterization of *Vibrio alginolyticus* isolated from diseased kuruma prawn, *Penaeus japonicus*. *Lett. Appl. Microbiol.* **22**, 111–114 (1996).
128. Le Moullac, G. *et al.* Effect of hypoxic stress on the immune response and the resistance to vibriosis of the shrimp *Penaeus stylirostris*. *Fish Shellfish Immunol.* **8**, 621–629 (1998).
129. Le Roux, F., Gay, M., Lambert, C., Waechter, M., Poubalanne, S., Chollet, B., Nicolas, J-L., Berthe, F. Comparative analysis of *Vibrio splendidus*-related strains isolated during *Crassostrea gigas* mortality events. *Aquat. Living Resourc.* **15**, 251-258 (2002).
130. Li, C.-C. & Chen, J.-C. The immune response of white shrimp *Litopenaeus vannamei* and its susceptibility to *Vibrio alginolyticus* under low and high pH stress. *Fish Shellfish Immunol.* **25**, 701–709 (2008).
131. Li, E., Lim, C., Klesius, P. & Cai, C. Enhancement effects of dietary wheat distiller's dried grains with solubles on growth, immunity, and resistance to *Edwardsiella ictaluri* challenge of channel catfish, *Ictalurus punctatus*. *J. World Aquac. Soc.* **43**, 814–827 (2012).
132. Li, Y. & Cai, S.-H. Identification and pathogenicity of *Aeromonas sobria* on tail-rot disease in juvenile tilapia *Oreochromis niloticus*. *Curr Microbiol* **62**, 623–627 (2011).
133. Lim, C., Klesius, P. H., Li, M. H. & Robinson, E. H. Interaction between dietary levels of iron and vitamin C on growth, hematology, immune response and resistance of channel catfish (*Ictalurus punctatus*) to *Edwardsiella ictaluri* challenge. *Aquaculture* **185**, 313–327 (2000).
134. Lim, C., Yildirim‑Aksoy, M. & Klesius, P. H. Growth response and resistance to *Edwardsiella ictaluri* of channel catfish, *Ictalurus punctatus*, fed diets containing distiller's dried grains with solubles. *J. World Aquac. Soc.* **40**, 182–193 (2009).
135. Liu, C.-H. & Chen, J.-C. Effect of ammonia on the immune response of white shrimp *Litopenaeus vannamei* and its susceptibility to *Vibrio alginolyticus*. *Fish Shellfish Immunol.* **16**, 321–334 (2004).
136. Liu, C.-H., Cheng, W., Hsu, J.-P. & Chen, J.-C. *Vibrio alginolyticus* infection in the white shrimp *Litopenaeus vannamei* confirmed by polymerase chain reaction and 16S rDNA sequencing. *Dis. Aquat. Org.* **61**, 169–174 (2004).
137. Liu, J. Y., Li, A. H., Zhou, D. R., Wen, Z. R. & Ye, X. P. Isolation and characterization of *Edwardsiella ictaluri* strains as pathogens from diseased yellow catfish *Pelteobagrus fulvidraco* (Richardson) cultured in China. *Aquac. Res.* **41**, 1835–1844 (2010).
138. Liu, J. Y. & Li, A. H. First case of *Aeromonas schubertii* infection in the freshwater cultured

snakehead fish, *Ophiocephalus argus* (Cantor), in China. *J. Fish Dis.* **35**, 335–342 (2012).
139. Liu, P.-C., Lin, J.-Y., Chuang, W.-H. & Lee, K.-K. Isolation and characterization of pathogenic *Vibrio harveyi* (*V. carchariae*) from the farmed marine cobia fish *Rachycentron canadum* L. with gastroenteritis syndrome. *World J. Microbiol. Biotechnol.* **20**, 495–499 (2004).
140. Liu, R. *et al.* Identification and characterisation of pathogenic *Vibrio splendidus* from Yesso scallop (*Patinopecten yessoensis*) cultured in a low temperature environment. *J. Invertebr. Pathol.* **114**, 144–150 (2013).
141. Liuxy, P.-C., Lee, K.-K. & Chen, S.-N. Pathogenicity of different isolates of *Vibrio harveyi* in tiger prawn, *Penaeus monodon*. *Letters in Applied Microbiology* **22**, 413–416 (1996).
142. Magnadóttir, B., Bambir, S. H., Gudmundsdóttir, B. K., Pilström, L. & Helgason, S. Atypical *Aeromonas salmonicida* infection in naturally and experimentally infected cod, *Gadus morhua* L. *J. Fish Dis.* **25**, 583–597 (2002).
143. Manning, B. B., Abbas, H. K., Wise, D. J. & Greenway, T. The effect of feeding diets containing deoxynivalenol contaminated corn on channel catfish (*Ictalurus punctatus*) challenged with *Edwardsiella ictaluri*. *Aquac. Res.* **45**, 1782–1786 (2014).
144. Manning, B. B., Wise, D. J., Abbas, H. K. & Peterson, B. C. Channel Catfish, *Ictalurus punctatus*, fed diets containing aflatoxin from moldy corn do not experience increased mortality after challenge with *Edwardsiella ictaluri*. *J. World Aquac. Soc.* **42**, 598–602 (2011).
145. Martins, M. L. *et al.* Isolation and experimental infection with *Vibrio alginolyticus* in the sea horse, *Hippocampus reidi* Ginsburg, 1933 (Osteichthyes: Syngnathidae) in Brazil. *Braz. J. Biol.* **70**, 205–209 (2010).
146. Matsuyama, T., Kamaishi, T., Ooseko, N., Kurohara, K. & Iida, T. Pathogenicity of motile and non-motile *Edwardsiella tarda* to some marine fish. *Fish Pathol.* **40**, 133–135 (2005).
147. Medina Félix, D. *et al.* Survival of *Litopenaeus vannamei* shrimp fed on diets supplemented with *Dunaliella sp.* is improved after challenges by *Vibrio parahaemolyticus*. *J. Invertebr. Pathol.* **148**, 118–123 (2017).
148. Meng, J., Zhang, L., Huang, B., Li, L. & Zhang, G. Comparative analysis of oyster (*Crassostrea gigas*) immune responses under challenge by different *Vibrio* strains and conditions. *Molluscan Res.* **35**, 1–11 (2015).
149. Mikulski, C.M., Burnett, L.E., Burnet, K.G. The effects of hypercapnic hypoxia on the survival of shrimp challenged with *Vibrio parahaemolyticus*. *J. Shellfish Res.* **19**, 301-311 (2000).
150. Mo, Z.-Q. *et al.* Outbreak of *Edwardsiella tarda* infection in farm-cultured giant mottled eel *Anguilla marmorata* in China. *Fish Sci.* **81**, 899–905 (2015).
151. Mohamad, S. & Abasali, H. Effect of plant extracts supplemented diets on immunity and resistance to *Aeromonas hydrophila* in common carp (*Cyprinus carpio*). *Agric. J.* **5**, 119–127 (2010).
152. Mohammed, H. H. & Arias, C. R. Protective efficacy of *Nigella sativa* seeds and oil against columnaris disease in fishes. *J. Fish Dis.* **39**, 693–703 (2016).
153. Mohanty, B. R. & Sahoo, P. K. Immune responses and expression profiles of some immune-related genes in Indian major carp, *Labeo rohita* to *Edwardsiella tarda* infection. *Fish Shellfish Immunol.* **28**, 613–621 (2010).
154. Monte, M., Urquhart, K., Secombes, C. J. & Collet, B. Individual monitoring of immune responses in rainbow trout after cohabitation and intraperitoneal injection challenge with *Yersinia ruckeri*. *Fish Shellfish Immunol.* **55**, 469–478 (2016).
155. Nagai, T. Nagai, T. Water temperature effect on *Edwardsiella ictaluri* infection of Ayu *Plecoglossus altivelis*. *Fish Pathol.* **49**, 61-63 (2014).
156. Ng, W.-K., Koh, C.-B., Teoh, C.-Y. & Romano, N. Farm-raised tiger shrimp, *Penaeus*

monodon, fed commercial feeds with added organic acids showed enhanced nutrient utilization, immune response and resistance to *Vibrio harveyi* challenge. *Aquaculture* **449**, 69–77 (2015).

157. Nguyen, T. V., Alfaro, A. C., Merien, F., Young, T. & Grandiosa, R. Metabolic and immunological , with *Vibrio sp. J. Invertebr. Pathol.* **157**, 80–89 (2018).
158. Nicolas, J. L., Basuyaux, O., Mazurié, J. & Thébault, A. *Vibrio carchariae*, a pathogen of the abalone *Haliotis tuberculata*. *Dis. Aquat. Org.* **50**, 35–43 (2002).
159. Nishimori, E., Hasegawa, O., Numata, T. & Wakabayashi, H. *Vibrio carchariae* causes mass mortalities in Japanese abalone, *Sulculus diversicolor supratexta*. *Fish Pathol*. **33**, 495-502 (1998).
160. Nya, E. J. & Austin, B. Use of dietary ginger, *Zingiber officinale* Roscoe, as an immunostimulant to control *Aeromonas hydrophila* infections in rainbow trout, Oncorhynchus mykiss (Walbaum). *J. Fish Dis.* **32**, 971–977 (2009).
161. Nya, E. J. & Austin, B. Use of garlic, *Allium sativum*, to control *Aeromonas hydrophila* infection in rainbow trout, *Oncorhynchus mykiss* (Walbaum). *J. Fish Dis.* **32**, 963–970 (2009).
162. Nya, E. J. & Austin, B. Development of immunity in rainbow trout (*Oncorhynchus mykiss*, Walbaum) to *Aeromonas hydrophila* after the dietary application of garlic. *Fish Shellfish Immunol.* **30**, 845–850 (2011).
163. Oh, M.-J. *et al.* Change of pathogenicity in Olive flounder *Paralichthys olivaceus* by co-infection of *Vibrio harveyi, Edwardsiella* tarda and marine birnavirus. *Aquaculture* **257**, 156–160 (2006).
164. Ohtani, M. *et al.* Effects of fish size and route of infection on virulence of a Danish *Yersinia ruckeri* O1 biotype 2 strain in rainbow trout (*Oncorhynchus mykiss*). *Aquaculture* **503**, 519–526 (2019).
165. Oraić, D., Zrnčić, S., Šoštarić, B. & et al. Occurrence of enteric redmouth disease in rainbow trout (*Oncorhynchus mykiss*) on farms in Croatia. *Acta Vet. Hung.* **50**, 283–291 (2002).
166. Park, Y. *et al.* Use of probiotics to enhance growth, stimulate immunity and confer disease resistance to *Aeromonas salmonicida* in rainbow trout (*Oncorhynchus mykiss*). *Aquac. Res.* **48**, 2672–2682 (2017).
167. Perera, R. P., Johnson, S. K. & Lewis, D. H. Epizootiological aspects of *Streptococcus iniae* affecting tilapia in Texas. *Aquaculture* **152**, 25–33 (1997).
168. Pérez, M., Fernandez, L., Rodriguez & Nieto, T. Differential susceptibility to furunculosis of turbot and rainbow trout and release of the furunculosis agent from furunculosis-affected fish. *Dis. Aquat. Org.* **26**, 133–137 (1996).
169. Peterson, B. C., Peatman, E., Ourth, D. D. & Waldbieser, G. C. Effects of a phytogenic feed additive on growth performance, susceptibility of channel catfish to *Edwardsiella ictaluri* and levels of mannose binding lectin. *Fish Shellfish Immunol.* **44**, 21–25 (2015).
170. Phiwsaiya, K. *et al.* A Natural *Vibrio parahaemolyticus* ΔpirAVp pirBVp+ mutant kills shrimp but produces neither PirVp toxins nor acute hepatopancreatic necrosis disease lesions. *Appl Environ Microbiol* **83**, (2017).
171. Prado, S., Dubert, J. & Barja, J. L. Characterization of pathogenic vibrios isolated from bivalve hatcheries in Galicia, NW Atlantic coast of Spain. Description of *Vibrio tubiashii* subsp. *europaeus* [corrected] subsp. nov. *Syst. Appl. Microbiol.* **38**, 26–29 (2015).
172. Prado, S., Romalde, J. L., Montes, J. & Barja, J. L. Pathogenic bacteria isolated from disease outbreaks in shellfish hatcheries. First description of *Vibrio neptunius* as an oyster pathogen. *Dis. Aquat. Org.* **67**, 209–215 (2005).
173. Pretto-Giordano, L.G., Müller, E.E., de Freitas, J.C., da Silva, V.G. Evaluation of the pathogenesis of *Streptococcus agalactiae* in Nile tilapia (*Oreochromis niloticus*). *Braz. Arch. Biol. Technol.* **53**, 87-92.
174. Pridgeon, J. W. & Klesius, P. H. Virulence of *Aeromonas hydrophila* to channel catfish

Ictalurus punctatus fingerlings in the presence and absence of bacterial extracellular products. *Dis. Aquat. Org.* **95**, 209–215 (2011).
175. Prieto, Z., Salirrosas, D., Arqueros, M. & Sánchez-Tuesta, L. El extracto de *Caesalpinia spinosa* inhibe la infección in vivo de *Flavobacterium columnare* en tilapia. *Scientia Agropecuaria* **9**, 215–221 (2018).
176. Punitha, S. M. J. *et al.* Immunostimulating influence of herbal biomedicines on nonspecific immunity in Grouper *Epinephelus tauvina* juvenile against *Vibrio harveyi* infection. *Aquac. Int.* **16**, 511–523 (2008).
177. Pylkkö, P., Pohjanvirta, T., Madetoja, J. & Pelkonen, S. Characterisation of atypical *Aeromonas salmonicida* infection in Arctic charr *Salvelinus alpinus* and European grayling *Thymallus thymallus*. *Dis. Aquat. Org.* **66**, 121–128 (2005).
178. Qin, L., Xu, J. & Wang, Y. G. Edwardsiellosis in farmed turbot, *Scophthalmus maximus* (L.), associated with an unusual variant of *Edwardsiella tarda*: a clinical, aetiological and histopathological study. *J. Fish Dis.* **37**, 103–111 (2014).
179. Rahman, M. H., Suzuki, S. & Kawai, K. The effect of temperature on *Aeromonas hydrophila* infection in goldfish, *Carassius auratus*. *J. Appl. Ichthyol.* **17**, 282–285 (2001).
180. Rahman. Tail and fin rot Ddsease of Indian major carp and climbing perch in Bangladesh. *J. Biol. Sci.* **10**, 800-804 (2010).
181. Rahmatullah, M. *et al.* Isolation and pathogenicity of *Streptococcus iniae* in cultured red hybrid tilapia in Malaysia. *J. Aquat. Anim. Health* **29**, 208–213 (2017).
182. Raida, M. K. & Buchmann, K. Development of adaptive immunity in rainbow trout, *Oncorhynchus mykiss* (Walbaum) surviving an infection with *Yersinia ruckeri*. *Fish Shellfish Immunol.* **25**, 533–541 (2008).
183. Raida, M. K., Holten-Andersen, L. & Buchmann, K. Association between *Yersinia ruckeri* infection, cytokine expression and survival in rainbow trout (*Oncorhynchus mykiss*). *Fish Shellfish Immunol.* **30**, 1257–1264 (2011).
184. Raida, M. K. & Buchmann, K. Innate immune response in rainbow trout (*Oncorhynchus mykiss*) against primary and secondary infections with *Yersinia ruckeri* O1. *Dev. Comp. Immunol.* **33**, 35–45 (2009).
185. Raida, M. K., Larsen, J. L., Nielsen, M. E. & Buchmann, K. Enhanced resistance of rainbow trout, *Oncorhynchus mykiss* (Walbaum), against *Yersinia ruckeri* challenge following oral administration of *Bacillus subtilis* and *B. licheniformis* (BioPlus2B). *J. Fish Dis.* **26**, 495–498 (2003).
186. Ransangan, J., Lal, T.M., Al-Harbi, A.H. Characterization and experimental infection of *Vibrio harveyi* isolated from diseased Asian seabass (*Lates calcarifer*). *Malaysian J. Microbiol.* **8,** 104-115 (2012).
187. Rasmussen-Ivey, C. R. *et al.* Classification of a hypervirulent *Aeromonas hydrophila* pathotype responsible for epidemic outbreaks in warm-water fishes. *Front Microbiol* **7**, 1615 (2016).
188. Rattanachaikunsopon P. & Phumkhachorn, P. Protective effect of clove oil-supplemented Fish Diets on experimental *Lactococcus garvieae* infection in tilapia. *Biosci. Biotechnol. Biochem.* **73**, 2085–2089 (2009a).
189. Rattanachaikunsopon, P. & Phumkhachorn, P. Potential of Chinese chive oil as a natural antimicrobial for controlling *Flavobacterium columnare* infection in Nile tilapia *Oreochromis niloticus. Fish. Sci.* **75**, 1431 (2009b).
190. Rattanachaikunsopon, P. & Phumkhachorn, P. Potential of cinnamon (*Cinnamomum verum*) oil to control *Streptococcus iniae* infection in tilapia (*Oreochromis niloticus*). *Fish. Sci.* **76**, 287–293 (2010).
191. Reichley, S. R. *et al.* Comparative susceptibility of channel catfish, *Ictalurus punctatus*; blue catfish, *Ictalurus furcatus*; and channel (♀) × blue (♂) hybrid catfish to *Edwardsiella piscicida*, *Edwardsiella tarda*, and *Edwardsiella anguillarum*. *J. World Aquac. Soc.* **49**,

197–204 (2018).
192. Rengpipat, S., Tunyanun, A., Fast, A. W., Piyatiratitivorakul, S. & Menasveta, P. Enhanced growth and resistance to *Vibrio* challenge in pond-reared black tiger shrimp *Penaeus monodon* fed a *Bacillus* probiotic. *Dis. Aquat. Org.* **55**, 169–173 (2003).
193. Reyes-Becerril, M., López-Medina, T., Ascencio-Valle, F. & Esteban, M. Á. Immune response of gilthead seabream (*Sparus aurata*) following experimental infection with *Aeromonas hydrophila*. *Fish Shellfish Immunol.* **31**, 564–570 (2011).
194. Richards, G. P., Watson, M. A., Needleman, D. S., Church, K. M. & Häse, C. C. Mortalities of Eastern and Pacific oyster larvae caused by the pathogens *Vibrio coralliilyticus* and *Vibrio tubiashii*. *Appl. Environ. Microbiol.* **81**, 292–297 (2015).
195. Robertson, P.A.W. *et al.* Experimental *Vibrio harveyi* infections in *Penaeus vannamei* larvae. *Dis. Aquat. Org.* **32,** 151-155 (1998).
196. Rodkhum, C., Kayansamruaj, P. & Pirarat, N. Effect of water temperature on susceptibility to *Streptococcus agalactiae* serotype Ia Infection in Nile Tilapia (*Oreochromis niloticus*). *Thai J. Vet. Med.* **41**, 309–314 (2011).
197. Roomiani, L., Ahmadi, S., Ghaeni, M. Immune response and disease resistance in the white shrimp, *Litopenaeus vannamei* induced by potential probiotic *Lactobacillus bulgaricus. Ankara Üniv. Vet. Fak. Derg.* **65**, 323-329 (2018).
198. Roux, F. L. *et al.* Comparative analysis of *Vibrio splendidus*-related strains isolated during *Crassostrea gigas mortality* events. *Aquat. Living Resour.* **15**, 251–258 (2002).
199. Saeidi, M. R., Adel, M., Caipang, C. M. A. & Dawood, M. A. O. Immunological responses and disease resistance of rainbow trout (*Oncorhynchus mykiss*) juveniles following dietary administration of stinging nettle (*Urtica dioica*). *Fish Shellfish Immunol.* **71**, 230–238 (2017).
200. Sainz, J.C., Maeda-Martínez, A.N. & Ascencio, F. Experimental vibriosis induction with *Vibrio alginolyticus* of larvae of the Catarina scallop (*Argopecten ventricosus =circularis*) (Sowerby II, 1842). *Microb. Ecol.* **35**, 188–192 (1998).
201. Sakai, T. *et al.* Mass mortality of cultured kuruma Prawn *Penaeus japonicus* caused by *Vibrio nigripulchritudo. Fish Pathol.* **42**, 141–147 (2007).
202. Sakai, T. *et al.* Outbreaks *of Edwardsiella ictaluri* infection in ayu *Plecoglossus altivelis* in Japanese rivers. *Fish Pathol.* **43**, 152–157 (2008).
203. Sarkar, M. J. A. & Rashid, M. M. Pathogenicity of the bacterial isolate *Aeromonas hydrophila* to catfishes, carps and perch. *J. Bangladesh Agric. Univ.* **10**, 157 (2012).
204. Sarker, J. & Faruk, M. a. R. Experimental infection of *Aeromonas hydrophila* in pangasius. *Progr. Agric.* **27**, 392–399 (2016).
205. Sawabe, T. *et al.* Mass mortality of Japanese abalone *Haliotis discus hannai* caused by *Vibrio harveyi* infection. *Microbes Environ.* **22**, 300–308 (2007).
206. Selvin, J. & Lipton, A. P. *Vibrio alginolyticus* associated with white spot disease of *Penaeus monodon*. *Dis. Aquat. Org.* **57**, 147–150 (2003).
207. Sharma, A. K. *et al.* Virulent *Aeromonas veronii* strain BLB-01 associated with mass mortality of *Clarias batrachus* (Linnaeus, 1758). *Int. J. Curr. Microbiol. App. Sci.* **6**, 3668-3681 (2017).
208. Shoemaker, C. A., Evans, J. J. & Klesius, P. H. Density and dose: factors affecting mortality of *Streptococcus iniae* infected tilapia (*Oreochromis niloticus*). *Aquaculture* **188**, 229–235 (2000).
209. Shoemaker, C. A., Martins, M. L., Xu, D.-H. & Klesius, P. H. Effect of *Ichthyophthirius multifiliis* parasitism on the survival, hematology and bacterial load in channel catfish previously exposed to *Edwardsiella ictaluri*. *Parasitol. Res.* **111**, 2223–2228 (2012).
210. Silva, B. C. da *et al.* Haemorrhagic septicaemia in the hybrid surubim (*Pseudoplatystoma corruscans×Pseudoplatystoma fasciatum*) caused by *Aeromonas hydrophila*. *Aquac. Res.* **43**, 908–916 (2012).

211. Sivaram, V. *et al.* Growth and immune response of juvenile greasy groupers (*Epinephelus tauvina*) fed with herbal antibacterial active principle supplemented diets against *Vibrio harveyi* infections. *Aquaculture* **237**, 9–20 (2004).
212. Smyrli, M. *et al. Aeromonas veronii* infection associated with high morbidity and mortality in farmed European seabass *Dicentrarchus labrax* in the Aegean Sea, Greece. *Fish Pathol.* **52**, 68–81 (2017).
213. Soffientino, B. *et al.* Infectious necrotizing enteritis and mortality caused by *Vibrio carchariae* in summer flounder *Paralichthys dentatus* during intensive culture. *Dis. Aquat. Org.* **38**, 201–210 (1999).
214. Soltani, M., Munday, B. L. & Burke, C. M. The relative susceptibility of fish to infections by *Flexibacter columnaris* and *Flexibacter maritimus. Aquaculture* **140**, 259–264 (1996).
215. Soto, E. *et al.* Laboratory-controlled Challenges of Nile tilapia (*Oreochromis niloticus*) with *Streptococcus agalactiae*: comparisons between immersion, oral, intracoelomic and intramuscular routes of infection. *J. Comp. Pathol.* **155**, 339–345 (2016).
216. Soto, E. *et al. Edwardsiella ictaluri* as the causative agent of mortality in cultured Nile tilapia. *J. Aquat. Anim. Health* **24**, 81–90 (2012).
217. Soto‐Rodriguez, S. A., Cabanillas‐Ramos, J., Alcaraz, U., Gomez‐Gil, B. & Romalde, J. L. Identification and virulence of *Aeromonas dhakensis*, *Pseudomonas mosselii* and *Microbacterium paraoxydans* isolated from Nile tilapia, *Oreochromis niloticus*, cultivated in Mexico. *J. Appl. Microbiol.* **115**, 654–662 (2013).
218. Soto-Rodriguez, S. A. *et al.* Virulence of *Vibrio harveyi* responsible for the 'Bright-red' Syndrome in the Pacific white shrimp *Litopenaeus vannamei*. *J.Invertebr. Pathol.* **109**, 307–317 (2012).
219. Ström-Bestor, M. *et al.* Introduction of *Yersinia ruckeri* biotype 2 into Finnish fish farms. *Aquaculture* **308**, 1–5 (2010).
220. Suanyuk, N. *et al.* Mortality and pathology of hybrid catfish, *Clarias macrocephalus* (Günther) × *Clarias gariepinus* (Burchell), associated with *Edwardsiella ictaluri* infection in southern Thailand. *J. Fish Dis.* **37**, 385–395 (2014).
221. Sun, Z. *et al.* The protein expression profile in hepatopancreas of scallop *Chlamys farreri* under heat stress and *Vibrio anguillarum* challenge. *Fish Shellfish Immunol.* **36**, 252–260 (2014).
222. Suomalainen, L.-R., Tiirola, M. A. & Valtonen, E. T. Effect of *Pseudomonas sp.* MT5 baths on *Flavobacterium columnare* infection of rainbow trout and on microbial diversity on fish skin and gills. *Dis. Aquat. Org.* **63**, 61–68 (2005a).
223. Suomalainen, L.-R., Tiirola, M. A. & Valtonen, E. T. Influence of rearing conditions on *Flavobacterium columnare* infection of rainbow trout, *Oncorhynchus mykiss* (Walbaum). *J. Fish Dis.* **28**, 271–277 (2005b).
224. Talpur, A. D. & Ikhwanuddin, M. Dietary effects of garlic (*Allium sativum*) on haemato-immunological parameters, survival, growth, and disease resistance against *Vibrio harveyi* infection in Asian sea bass, *Lates calcarifer* (Bloch). *Aquaculture* **364–365**, 6–12 (2012).
225. Talpur, A. D. & Ikhwanuddin, M. *Azadirachta indica* (neem) leaf dietary effects on the immunity response and disease resistance of Asian seabass, *Lates calcarifer* challenged with *Vibrio harveyi*. *Fish Shellfish Immunol.* **34**, 254–264 (2013).
226. Tavares, G. C. *et al.* Disease outbreaks in farmed Amazon catfish (*Leiarius marmoratus x Pseudoplatystoma corruscans*) caused by *Streptococcus agalactiae*, *S. iniae*, and *S. dysgalactiae. Aquaculture* **495**, 384–392 (2018).
227. Thinh, N. H. *et al.* Combined immersion and oral vaccination of Vietnamese catfish (*Pangasianodon hypophthalmus*) confers protection against mortality caused by *Edwardsiella ictaluri*. *Fish Shellfish Immunol.* **27**, 773–776 (2009).
228. Tien, N. T., Dung, T. T., Tuan, N. A. & Crumlish, M. First identification of *Flavobacterium*

columnare infection in farmed freshwater striped catfish *Pangasianodon hypophthalmus*. *Dis. Aquat. Org.* **100**, 83–88 (2012).

229. Torrecillas, S. *et al.* Effects on mortality and stress response in European sea bass, *Dicentrarchus labrax* (L.), fed mannan oligosaccharides (MOS) after *Vibrio anguillarum* exposure. *J. Fish Dis.* **35**, 591–602 (2012).
230. Tran, L. *et al.* Determination of the infectious nature of the agent of acute hepatopancreatic necrosis syndrome affecting penaeid shrimp. *Dis. Aquat. Org.* **105**, 45–55 (2013).
231. Travers, M.-A. *et al.* Influence of temperature and spawning effort on *Haliotis tuberculata* mortalities caused by *Vibrio harveyi*: an example of emerging vibriosis linked to global warming. *Glob. Change Biol.* **15**, 1365–1376 (2009).
232. Tseng, I.-T. & Chen, J.-C. The immune response of white shrimp *Litopenaeus vannamei* and its susceptibility to *Vibrio alginolyticus* under nitrite stress. *Fish Shellfish Immunol.* **17**, 325–333 (2004).
233. Vagnes, O., Biering, E., Almas, K., Development of injection and cohabitation challenge models for atypical *Aeromonas salmonicida* in farmed ballan wrasse (*Labrus bergylta*). Veterinærinstituttets rapportserie 1-2014. Norwegian Veterinary Institute (2014).
234. Van Doan, H., Hoseinifar, S. H., Tapingkae, W. & Khamtavee, P. The effects of dietary kefir and low molecular weight sodium alginate on serum immune parameters, resistance against Streptococcus agalactiae and growth performance in Nile tilapia (*Oreochromis niloticus*). *Fish Shellfish Immunol.* **62**, 139–146 (2017).
235. Vaseeharan, B. & Ramasamy, P. Control of pathogenic *Vibrio spp.* by *Bacillus subtilis* BT23, a possible probiotic treatment for black tiger shrimp *Penaeus monodon*. *Lett. Appl. Microbiol.* **36**, 83–87 (2003).
236. Verma, D. K., Rathore, G., Pradhan, P. K., Sood, N. & Punia, P. Isolation and characterization of *Flavobacterium columnare* from freshwater ornamental goldfish *Carassius auratus*. *J Environ Biol* **36**, 433–439 (2015).
237. Verma, D. K. & Rathore, G. Molecular characterization of *Flavobacterium columnare* isolated from a natural outbreak of columnaris disease in farmed fish, *Catla catla* from India. *J. Gen. Appl. Microbiol.* **59**, 417–424 (2013).
238. Verner-Jeffreys, D. W. *et al.* Characterisation of a serotype O1 *Yersinia ruckeri* isolate from the Isle of Man: further evidence that O antigen serotype is not a reliable indicator of virulence. *Bull. Eur. Ass. Fish Pathol.* **31**, 86–91 (2011).
239. Volpatti, D., Chiara, B., Francesca, T. & Marco, G. Growth parameters, innate immune response and resistance to *Listonella* (*Vibrio*) *anguillarum* of *Dicentrarchus labrax* fed carvacrol supplemented diets. *Aquac. Res.* **45**, 31–44 (2013).
240. Wang, L.-U. & Chen, J.-C. The immune response of white shrimp *Litopenaeus vannamei* and its susceptibility to *Vibrio alginolyticus* at different salinity levels. *Fish Shellfish Immunol.* **18**, 269–278 (2005).
241. Wang, X. *et al.* Immune response and energy metabolism of *Chlamys farreri* under *Vibrio anguillarum* challenge and high temperature exposure. *Fish Shellfish Immunol.* **33**, 1016–1026 (2012).
242. Wassif, I.M. Biochemical and molecular characterization of *Aeromonas* species isolated from fish. *Alexandria J. Vet. Sci.* **57,** 38-45 (2018).
243. Welch, T. J. & Wiens, G. D. Construction of a virulent, green fluorescent protein-tagged *Yersinia ruckeri* and detection in trout tissues after intraperitoneal and immersion challenge. *Dis. Aquat. Org.* **67**, 267–272 (2005).
244. Welker, T. L., Shoemaker, C. A., Arias, C. R. & Klesius, P. H. Transmission and detection of *Flavobacterium columnare* in channel catfish *Ictalurus punctatus*. *Dis. Aquat. Org.* **63**, 129–138 (2005).
245. Wendling, C. C. & Wegner, K. M. Relative contribution of reproductive investment, thermal stress and *Vibrio* infection to summer mortality phenomena in Pacific oysters.

Aquaculture **412–413**, 88–96 (2013).
246. Wiedenmayer, A. A., Evans, J. J. & Klesius, P. H. Experimental *Edwardsiella tarda* infection in nonabraded channel catfish Ictalurus punctatus by immersion. *Fish. Sci.* **72**, 1124–1126 (2006).
247. Wiens, G. D. & Vallejo, R. L. Temporal and pathogen-load dependent changes in rainbow trout (*Oncorhynchus mykiss*) immune response traits following challenge with biotype 2 *Yersinia ruckeri*. *Fish Shellfish Immunol.* **29**, 639–647 (2010).
248. Wiklund, T. Virulence of atypical *Aeromonas salmonicida* isolated from ulcerated flounder *Platichthys flesus*. *Dis. Aquat. Org.* **21**, 145–150 (1995).
249. Won, K. M. & Park, S. I. Pathogenicity of *Vibrio harveyi* to cultured marine fishes in Korea. *Aquaculture* **285**, 8–13 (2008).
250. Wu, C.-C. *et al.* Dietary administration of *Gynura bicolor* (Roxb. Willd.) DC water extract enhances immune response and survival rate against *Vibrio alginolyticus* and white spot syndrome virus in white shrimp *Litopeneaus vannamei*. *Fish Shellfish Immunol.* **42**, 25–33 (2015).
251. Xia, Y. *et al.* Effects of dietary *Lactobacillus rhamnosus* JCM1136 and *Lactococcus lactis* subsp. *lactis* JCM5805 on the growth, intestinal microbiota, morphology, immune response and disease resistance of juvenile Nile tilapia, *Oreochromis niloticus*. *Fish Shellfish Immunol.* **76**, 368–379 (2018).
252. Xiao, J. *et al.* Isolation and identification of fish pathogen *Edwardsiella tarda* from mariculture in China. *Aquac. Res.* **40**, 13–17 (2008).
253. Xu, D.-H., Shoemaker, C. A. & LaFrentz, B. R. Enhanced susceptibility of hybrid tilapia to *Flavobacterium columnare* after parasitism by *Ichthyophthirius multifiliis*. *Aquaculture* **430**, 44–49 (2014).
254. Xu, D.-H., Shoemaker, C. A., Martins, M. L., Pridgeon, J. W. & Klesius, P. H. Enhanced susceptibility of channel catfish to the bacterium *Edwardsiella ictaluri* after parasitism by *Ichthyophthirius multifiliis*. *Vet. Microbiol.* **158**, 216–219 (2012).
255. Yang, R. *et al.* Pathogenesis and pathological analysis of *Edwardsiella tarda* from Dabry's sturgeon (*Acipenser dabryanus*) in China. *Aquaculture* **495**, 637–642 (2018).
256. Ye, S., Li, H., Qiao, G. & Li, Z. First case of *Edwardsiella ictaluri* infection in China farmed yellow catfish *Pelteobagrus fulvidraco*. *Aquaculture* **292**, 6-10 (2009).
257. Yılmaz, S., Ergun, S., Çelik, E. Ş. & Yigit, M. Effects of dietary humic acid on growth performance, haemato-immunological and physiological responses and resistance of rainbow trout, *Oncorhynchus mykiss* to *Yersinia ruckeri*. *Aquac. Res.* **49**, 3338–3349 (2018).
258. Yu, J., Koo, B. H., Kim, D. H., Kim, D. W. & Park, S. W. *Aeromonas sobria* infection in farmed mud loach (*Misgurnus mizolepis*) in Korea, a bacteriological survey. *Iran J. Vet. Res.* **16**, 194–201 (2015).
259. Yu, J.-H., Han, J.J., Kim, H.J., Kang, S.G., Park, S.W. First report of *Aeromonas veronii* infection in farmed Israeli carp *Cyprinus carpio* in Korea. *J. Fish. Pathol.* **23**, 165-176.
260. Yu, J.-H., Han, J. J., Park, K. S., Park, K. H. & Park, S. W. *Edwardsiella tarda* infection in Korean catfish, *Silurus asotus*, in a Korean fish farm. *Aquac. Res.* **41**, 19–26 (2009).
261. Yuasa, K., Kholidin, E. B., Panigoro, N. & Hatai, K. First isolation of *Edwardsiella ictaluri* from cultured striped catfish *Pangasius hypophthalmus* in Indonesia. *Fish Pathol.* **38**, 181–183 (2003).
262. Yue, X., Liu, B. & Sun, L. Isolation and characterization of a virulent *Vibrio sp*. bacterium from clams (*Meretrix meretrix*) with mass mortality. *J. Invertebr. Pathol.* **106**, 242–249 (2011).
263. Yue, X., Liu, B., Xiang, J. & Jia, J. Identification and characterization of the pathogenic effect of a *Vibrio parahaemolyticus*-related bacterium isolated from clam *Meretrix meretrix* with mass mortality. *J. Invertebr. Pathol.* **103**, 109–115 (2010).

264. Zaefarian, A., Yeganeh, S. & Adhami, B. Dietary effects of garlic powder (*Allium sativum*) on growth, blood indices, carcass composition, and lysozyme activity in brown trout (*Salmo caspius*) and resistance against *Yersinia ruckeri* infection. *Aquacult Int.* **25**, 1987–1996 (2017).
265. Zahran, E., Abd El-Gawad, E. A. & Risha, E. Dietary *Withania somnifera* root confers protective and immunotherapeutic effects against *Aeromonas hydrophila* infection in Nile tilapia (*Oreochromis niloticus*). *Fish Shellfish Immunol.* **80**, 641–650 (2018).
266. Zeraatpisheh, F., Firouzbakhsh, F. & Khalili, K. J. Effects of the macroalga *Sargassum angustifolium* hot water extract on hematological parameters and immune responses in rainbow trout (*Oncohrynchus mykiss*) infected with *Yersinia rukeri*. *J Appl Phycol* **30**, 2029–2037 (2018).
267. Zhang, D. *et al.* Impact of oral and waterborne administration of rhamnolipids on the susceptibility of channel catfish (Ictalurus punctatus) to *Flavobacterium columnare* infection. *Fish Shellfish Immunol.* **60**, 44–49 (2017).
268. Zhang, D., Moreira, G. S. A., Shoemaker, C., Newton, J. C. & Xu, D.-H. Detection and quantification of virulent *Aeromonas hydrophila* in channel catfish tissues following waterborne challenge. *FEMS Microbiol. Lett.* **363**, (2016).
269. Zhang, D., Xu, D.-H. & Shoemaker, C. Experimental induction of motile *Aeromonas* septicemia in channel catfish (*Ictalurus punctatus*) by waterborne challenge with virulent *Aeromonas hydrophila*. *Aquac. Rep.* **3**, 18–23 (2016).
270. Zhao, H. *et al.* Impact of feed additives on surface mucosal health and columnaris susceptibility in channel catfish fingerlings, *Ictalurus punctatus*. *Fish Shellfish Immunol.* **46**, 624–637 (2015).
271. Zheng, Z. L. *et al.* Evaluation of oregano essential oil (*Origanum heracleoticum* L.) on growth, antioxidant effect and resistance against *Aeromonas hydrophila* in channel catfish (*Ictalurus punctatus*). *Aquaculture* **292**, 214–218 (2009).
272. Zheng, W., Cao, H., Yang, X. *Aeromonas veronii* infection in the cultured snakehead fish, *Ophiocephalus argus* (Cantor). *African J. Microbiol. Res.* ***6,*** 7218 – 7223 (2012).
273. Zhou, J. *et al.* A nonluminescent and highly virulent *Vibrio harveyi* strain is associated with 'Bacterial White Tail Disease' of *Litopenaeus vannamei* shrimp. *PLOS ONE* **7**, e29961 (2012).
