# Supplementary material for: Aquaculture at the crossroads of global warming and antimicrobial resistance
Source: Nat Commun. 2020 Apr 20;11:1870. doi: 10.1038/s41467-020-15735-6 (PMC7170852; doi:10.1038/s41467-020-15735-6)
Supplement: Supplementary file 5 — Supplementary Data 2 [file 41467_2020_15735_MOESM5_ESM.pdf]

Supplementary data 2: References used in the dataset that investigated the antimicrobial resistance from aquaculture-related bacteria and that was used to calculate the Multi-Antibiotic Index (MAR) for 40 countries.


1. Abdel-Aziz, M., Eissa, A. E., Hanna, M. & Okada, M. A. Identifying some pathogenic *Vibrio/Photobacterium* species during mass mortalities of cultured Gilthead seabream (*Sparus aurata*) and European seabass (*Dicentrarchus labrax*) from some Egyptian coastal provinces. *Int. J. Vet. Sci. Med.* **1**, 87–95 (2013).
2. Abdella, B., El-Wazzan, E., El-Sersy, N. A., Sabry, S. A. & El-Helow, E. R. Pathogenicity and antibiotic susceptibility of two bacterial pathogens associated with the clam *Tapes decussatus* in some Egyptian fisheries. *Ege J. Fish. Aquat. Sci.* **34**, 383-389 (2017).
3. Abu-Elala, N., Abdelsalam, M., Marouf, S. & Setta, A. Comparative analysis of virulence genes, antibiotic resistance and gyrB-based phylogeny of motile *Aeromonas* species isolates from Nile tilapia and domestic fowl. *Lett. App. Microbiol.* **61**, 429–436 (2015).
4. Afizi, M. S. K., Fatimah, B. S. S., Mariana, N. S. & Abdel-Hadi, Y. M. Herbal and antibiotic resistance of *Aeromonas* bacteria isolated from cultured fish in Egypt and Malaysia. *J. Fish. Aquat. Sci.* **8,** 425-429 (2013).
5. *Aisyhah*, M. S., Amal, M. N. A., Zamri-Saad, M., Siti-Zahrah, A. & Shaqinah, N. N. *Streptococcus agalactiae* isolates from cultured fishes in Malaysia manifesting low resistance pattern towards selected antibiotics. *J. Fish Dis.* **38**, 1093–1098 (2015).
6. Al-Sunaiher, A. E., Ibrahim, A. S. S. & Al-Salamah, A. A. Association of *Vibrio* species with disease incidence in some cultured fishes in the kingdom of Saudi Arabia. *W. Appl. Sci. J.* **8**, 653-660 (2010).
7. Albuquerque-Costa, R., Araújo, R. L., Souza, O. V. & Vieira, R. H. S. Antibiotic-resistant Vibrios in farmed shrimp. *Biomed. Res. Int.* **2015**, 505914 (2015).
8. Ali, S. et al. Identification, characterization and antibiotic sensitivity of *Aeromonas hydrophila*, a causative agent of epizootic ulcerative syndrome in wild and farmed fish from Potohar, Pakistan. *Pakistan J. Zool.* **48**, 899–901 (2016).
9. Altun, S., Onuk, E. E., ÇİftçĪ, A., BüyükekĪz, A. G. & Duman, M. Phenotypic, genotypic characterisation and antimicrobial susceptibility determination of *Lactococcus garvieae* strains. *Kafkas Üniv. Vet. Fak. Derg.* **19**, 375–381 (2013).
10. Amal, M. N. A. et al. An outbreak of *Streptococcus agalactiae* infection in cage-cultured golden pompano, *Trachinotus blochii* (Lacépède), in Malaysia. *J. Fish Dis.* **35**, 849–852 (2012).
11. Amiri, A. A., Amiri, S., Behrouzi, S., Naseri, F., Arabzadeh, P., Babaalian, A. & Khodadadi, A. Survey of the sensitivity of *Vibrio* spp. isolated from *Litopenaeus vannamei* to different antibiotics. *J. Fish. Aquat. Sci.* **9**, 487-495 (2014).
12. Ansari, M. & Raissy, M. *In vitro* susceptibility of commonly used antibiotics against *Vibrio* spp. isolated from lobster (*Panulirus homarus*). *African J Microbiol. Res.* **4**, 2629-2631(2010).
13. Anyanwy, M. U., Chah, K. F. & Shoyinka, V. S. Antibiogram of aerobic bacteria isolated from skin lesions of African catfish cultured in Southeast Nigeria. *Int. J. Fish. Aquat. Stud.* **2**, 134-141 (2014).
14. Balta, F. Phenotypic, serotypic and genetic characterization and antimicrobial susceptibility determination of *Vibrio anguillarum,* isolated from cultured sea bass (*Dicentrarchus labrax* L., 1758) in the Southeast Black Sea, Turkey. *Fresenius Env. Bull.* **25**, 4393-4400 (2016).
15. Balta, F., Sandalli, C., Kayis, S. & Ozgumus, O.B. Molecular analysis of antimicrobial resistance in *Yersinia ruckeri* strains isolated from rainbow trout *(Onorhynchus mykiss)* grown in commercial fish farms in Turkey. *Bull. Eur. Ass. Fish. Pathol.* **30**, 211-219 (2010).
16. Banerjee, S., Ooi, M. C., Shariff, M. & Khatoon, H. Antibiotic resistant *Salmonella* and *Vibrio* associated with farmed *Litopenaeus vannamei. Sci. World J.* **2012**, 130136 (2012).

17. Bharathkumar, G. & Abraham, T. J. Antibiotic susceptibility of Gram-negative bacteria isolated from freshwater fish hatcheries of West Bengal, India*. Indian J. Fish.* **58**, 135-138 (2011).
18. Borty, S. C. et al. Isolation, molecular identification and antibiotic susceptibility profile of *Aeromonas hydrophila* from cultured indigenous Koi (*Anabus testudineus*) of Bangladesh. *Asian J. Med. Biol. Res.* **2**, 332–340 (2016).
19. Brouwer, M. S. M. et al. *Enterobacter cloacae* complex isolated from shrimps from Vietnam carrying blaIMI-1 resistant to carbapenems but not cephalosporins. *Antimicrob. Agents Chemother.* **62**, (2018).
20. Calvez, S., Gantelet, H., Blanc, G., Douet, D.-G. & Daniel, P. *Yersinia ruckeri* biotypes 1 and 2 in France: presence and antibiotic susceptibility. *Dis. Aquat. Org.* **109**, 117–126 (2014).
21. Capkin, E., Ozdemir, S., Ozturk, R. C. & Altinok, I. Determination and transferability of plasmid-mediated antibiotic resistance genes of the bacteria isolated from rainbow trout. *Aquac. Res.* **48**, 5561–5575 (2017).
22. Capkin, E., Terzi, E. & Altinok, I. Occurrence of antibiotic resistance genes in culturable bacteria isolated from Turkish trout farms and their local aquatic environment. *Dis. Aquat. Org.* **114**, 127–137 (2015).
23. Carnevia, D., Letamendia, M. & Perretta, A. Pathogenic Gram-negative bacteria isolated from ornamental fish in Uruguay: characterization and antibiotic resistance. *Bull. Eur. Ass. Fish Pathol*. **33**, 181-186 (2013).
24. Carriero, M. M., Mendes-Maia A. A., Moro-Sousa, R. L. & Henrique-Silva, F. Characterization of a new strain of *Aeromonas dhakensis* isolated from diseased pacu fish (*Piaractus mesopotamicus*) in Brazil. *J. Fish Dis.* **39**, 1285–1295 (2016).
25. Chandrarathna, H. P. S. U. et al. Outcome of co-infection with opportunistic and multidrug resistant *Aeromonas hydrophila* and *A. veronii* in zebrafish: Identification, characterization, pathogenicity and immune responses. *Fish Shellfish Immunol.* **80**, 573–581 (2018).
26. Chen, J., Ding, X., Zhu, N., Kong, L. & He, Z. Prevalence and antimicrobial susceptibility of *Aeromonas* species from diseased Chinese soft-shelled turtles (*Trionyx sinens*). *Aquac. Res.* **46**, 1527–1536 (2015).
27. Chideroli, R. T. *et al.* Emergence of a new multidrug-resistant and highly virulent serotype of *Streptococcus agalactiae* in fish farms from Brazil. *Aquaculture* **479**, 45–51 (2017).
28. Chikwendu, C. I., Ibe, S. N. & Okpokwasili, G. C. Multiple antimicrobial resistance in *Vibrio* spp. isolated from river and aquaculture water sources in Imo State, Nigeria. *British Microbiol. Res. J.* **4**, 560–569 (2014).
29. Cízek, A. et al. Antimicrobial resistance and its genetic determinants in aeromonads isolated in ornamental (koi) carp (*Cyprinus carpio* koi) and common carp (*Cyprinus carpio*). *Vet. Microbiol.* **142**, 435–439 (2010).
30. Dadar, M., Adel, M. & Zorriehzahra, M. J. Isolation and phylogenic analysis of emerging new antibiotic resistance bacteria, *Acinetobacter lwoffii*, associated with mortality in farmed rainbow trout. *Iranian J. Fish. Sci*. **15**, 1279-1292 (2016).
31. Dahanayake, P. S., Silva, B. C. J. D., Hossain, S., Shin, G.-W. & Heo, G.-J. Occurrence, virulence factors, and antimicrobial susceptibility patterns of *Vibrio* spp. isolated from live oyster (*Crassostrea gigas*) in Korea. *J. Food Saf.* **38**, e12490 (2018).
32. Dahdouh, B., Basha, O., Khalil, S. & Tanekhy, M. Molecular characterization, antimicrobial susceptibility and salt tolerance of *Aeromonas hydrophila* from fresh, brackish and marine fishes. *Alex. J. Vet. Sci.* **48**, 46–53 (2016).
33. Dangwetngam, M., Suanyuk, N., Kong, F. & Phromkunthong, W. Serotype distribution and antimicrobial susceptibilities of *Streptococcus agalactiae* isolated from infected cultured tilapia (*Oreochromis niloticus*) in Thailand: Nine-year perspective. *J. Med. Microbiol.* **65**, 247–254 (2016).
34. Daood, N. Isolation and antibiotic susceptibility of *Aeromonas* spp. from freshwater fish farm and farmed carp (Dam of 16 Tishreen, Lattakia). *Damascus Univ. J. Basic Sci*. **28**,

27-39 (2012).
35. Das, A., Saha, D. & Pal, J. Antimicrobial resistance and in vitro gene transfer in bacteria isolated from the ulcers of EUS-affected fish in India. *Lett. Appl. Microbiol.* **49**, 497–502 (2009).
36. Deng, M. et al. Outbreaks of Streptococcosis associated with *Streptococcus iniae* in Siberian sturgeon (*Acipenser baerii*) in China. *Aquacult. Res.* **48**, 909–919 (2017).
37. Deng, Y.-T. et al. Analysis of antimicrobial resistance genes in *Aeromonas* spp. isolated from cultured freshwater animals in China. *Microb. Drug Resist.* **20**, 350–356 (2014).
38. Dubert, J., Romalde, J. L., Prado, S. & Barja, J. L. *Vibrio bivalvicida* sp. nov., a novel larval pathogen for bivalve molluscs reared in a hatchery. *Syst. App. Microbiol.* **39**, 8–13 (2016).
39. Dung, T. T. et al. IncK plasmid-mediated tetracycline resistance in *Edwardsiella ictaluri* isolates from diseased freshwater catfish in Vietnam. *Aquaculture* **295**, 157–159 (2009).
40. Durmaz, Y., Onuk, E. E. & Ciftci, A. Investigation of the presence and antibiotic susceptibilities of *Flavobacterium psychrophilum* in rainbow trout farms *(Oncorhynchus mykiss* Walbaum, 1792) in the Middle and Eastern Black Sea Regions of Turkey. *Ankara Üniv. Vet. Fak. Derg.* **59,** 141-146 (2012).
41. Efuntoye, M. O., Olurin, K. B. & Jegede, G. C. Bacterial flora from healthy *Clarias gariepinus* and their antimicrobial resistance pattern. *Adv. J. Food Sci. Technol.* **4**, 121-125 (2012).
42. El-Barbary, M. I. & Hal, A. M. Isolation and molecular characterization of some bacterial pathogens in El-Serw fish farm, Egypt. *Egyptian J. Aquat. Biol. Fish.* **20**, 115–127 (2016).
43. El-Barbary, M. I. & Hal, A. M. Molecular identification and pathogenicity of *Citrobacter* and *Serratia* species isolated from cultured *Oreochromis niloticus*. *Egyptian J. Aquat. Res.* **43**, 255–263 (2017).
44. El-Far, S. A. H., Khalil, R. H., Saad, T. T., El-Tanekhy, M. & Abdel-Latif H. M. R. Occurrence, characterization and antibiotic resistance patterns of bacterial communities encountered in mass kills of pond cultured Indian prawn (*Fenneropenaeus indicus*) at Damietta governorate, Egypt. *Int. J. Fish. Aquat. Studies* **2**, 271-276 (2015).
45. Elmahdi, S. et al. *Vibrio parahaemolyticus* and *Vibrio vulnificus* recovered from oysters during an oyster Relay Study. *Appl. Environ. Microbiol.* **84**, (2018).
46. Eshik, M. M. E., Abedin, M. M., Punom, N. J., Begum, M. K. & Rahman, M. S. Molecular identification of AHPND positive *Vibrio Parahaemolyticus* causing an outbreak in South-West Shrimp Farming Regions of Bangladesh. *J. Bangladesh Acad. Sci.* **41**, 127–135 (2017).
47. Fernández-Alarcón, C. et al. Detection of the floR Gene in a Diversity of florfenicol resistant Gram-Negative bacilli from freshwater salmon farms in Chile. *Zoonoses Public Health* **57**, 181–188 (2010).
48. Fernández-Álvarez, C., Gijón, D., Álvarez, M. & Santos, Y. First isolation of *Aeromonas salmonicida* subspecies *salmonicida* from diseased sea bass, *Dicentrarchus labrax* (L.), cultured in Spain. *Aquacult. Rep.* **4**, 36–41 (2016).
49. Fri, J., Ndip, R. N., Njom, H. A. & Clarke, A. M. Antibiotic susceptibility of non-cholera *Vibrios* isolated from farmed and wild marine fish (*Argyrosomus japonicus*), implications for public health. *Microb. Drug Resist.* **24**, 1296–1304 (2018).
50. Gashgari, R. M. & Selim, S. A. Detection and characterization of antimicrobial resistance and putative virulence genes in *Aeromonas veronii* biovar *sobria* isolated from gilthead sea bream (*Sparus aurata* L.). *Foodborne Pathog. Dis.* **12**, 806–811 (2015).
51. Geng, Y. et al. Isolation and characterization of *Edwardsiella ictaluri* from southern catfish, *Silurus soldatovi meridionalis*, (Chen) cultured in China. *J. World Aquac. Soc.* **44**, 273–281 (2013).
52. Geng, Y., Wang, K., Chen, D., Fan, F. & Huang, Y. Isolation and characterization of *Edwardsiella ictaluri* from cultured yellow catfish (*Pelteobagrus fulvidraco*). *Israeli J. Aquac.* **62**, 105-115 (2010).

53. Han, J. E., Mohney, L. L., Tang, K. F. J., Pantoja, C. R. & Lightner, D. V. Plasmid mediated tetracycline resistance of *Vibrio parahaemolyticus* associated with acute hepatopancreatic necrosis disease (AHPND) in shrimps. *Aquac. Rep.* **2**, 17–21 (2015).
54. Hardi, E. H. et al. Identification of potentially pathogenic bacteria from tilapia (*Oreochromis niloticus*) and channel catfish (*Clarias batrachus*) culture in Samarinda, East Kalimantan, Indonesia. *Biodiversitas* **19**, 480–488 (2018).
55. Hashem, M. & El-Barbary, M. *Vibrio harveyi* infection in Arabian Surgeon fish (*Acanthurus sohal*) of Red Sea at Hurghada, Egypt. *Egyptian J. Aquat. Res.* **39**, 199–203 (2013).
56. Hassan, M. A., Noureldin, E. A., Mahmoud, M. A. & Fita, N. A. Molecular identification and epizootiology of *Aeromonas veronii* infection among farmed *Oreochromis niloticus* in Eastern Province, KSA. *Egyptian J. Aquat. Res.* **43**, 161–167 (2017).
57. Heenatigala, P. P. M. & Fernando, M. U. L. Occurrence of bacteria species responsible for vibriosis in shrimp pond culture systems in Sri Lanka and assessment of the suitable control measures. *Sri Lanka J. Aquat. Sci.* **21**, 1–17 (2016).
58. Henríquez, P., Kaiser, M., Bohle, H., Bustos, P. & Mancilla, M. Comprehensive antibiotic susceptibility profiling of Chilean *Piscirickettsia salmonis* field isolates. *J. Fish Dis.* **39**, 441–448 (2016).
59. Hossain, M. S., Aktaruzzaman, M., Fakhruddin, A. N. M., Uddin, M. J., Rahman, S. H., Chowdhury, M. A. Z. & Alam, M. K. Prevalence of multiple drug resistant pathogenic bacteria in cultured black tiger shrimp (*Penaeus monodon* Fabricius). *Glob. J. Env. Res.* **6,** 118-124 (2012).
60. Hua, L. M. & Apun, K. Antimicrobial susceptibilities of *Vibrio parahaemolyticus* isolates from tiger shrimps (*Penaeus monodos*) aquaculture in Kuching, Sarawak. *Res. J. Microbiol.* ***8***, 55-62 (2013).
61. Igbinosa, E. O. Detection and antimicrobial resistance of *Vibrio* isolates in aquaculture environments: implications for public health. *Microb. Drug Resist.* **22**, 238–245 (2016).
62. Jagoda, S. S. et al. Characterization and antimicrobial susceptibility of motile aeromonads isolated from freshwater ornamental fish showing signs of septicaemia. *Dis. Aquat. Org.* **109**, 127–137 (2014).
63. Jayasree, L., Janakiram, P., Madhavi, R. & Saibab, P. Multiple antibiotic resistance pattern of *Vibrio harveyi* from luminous vibriosis affected cultured tiger shrimp, *Penaeus monodon* in Andhra Pradesh, India. Int. J. Curr. Microbiol. App. Sci. **4**, 523-535 (2015).
64. Jeeva, S., Lekhsmi, N. C. J. P., Brindha, J. R. & Vasudevan, A. Studies on antibiotic susceptibility of *Aeromonas hydrophila* isolated from goldfish (*Carassius auratus*). *Int. J. Curr. Microbiol. Appl. Sci.* **2**, 7-13 (2013).
65. Jun, J. W., Kim, Y. H., Gomez, D. K., Choresca, C. H., Han, J. E., Shin, S. P. & Park, C. S. Occurrence of tetracycline-resistance *Aeromonas hydrophila* infection in Korean cyprinid loach *(Misgurnus anguillicaudatus*). *African J. Microbiol. Res*. **4**, 849-855 (2010).
66. Kang, C.-H. et al. Characterization of *Vibrio parahaemolyticus* isolated from oysters in Korea: Resistance to various antibiotics and prevalence of virulence genes. *Mar. Pollut. Bull.* **118**, 261–266 (2017).
67. Kang, C.-H. *et al.* Prevalence and antimicrobial susceptibility of *Vibrio parahaemolyticus* isolated from oysters in Korea. *Environ. Sci. Pollut. Res. Int.* **23**, 918–926 (2016).
68. Kang, C.-H., Shin, Y., Yu, H., Kim, S. & So, J.-S. Antibiotic and heavy-metal resistance of *Vibrio parahaemolyticus* isolated from oysters in Korea. *Mar. Pollut. Bull.* **135**, 69–74 (2018).
69. Kannika, K. et al. Molecular serotyping, virulence gene profiling and pathogenicity of *Streptococcus agalactiae* isolated from tilapia farms in Thailand by multiplex PCR. *J. Appl. Microbiol.* **122**, 1497–1507 (2017).
70. Kawanishi, M. et al. Drug resistance and pulsed-field gel electrophoresis patterns of *Lactococcus garvieae* isolates from cultured *Seriola* (yellowtail, amberjack and kingfish) in Japan. *Lett. Appl. Microbiol.* **40**, 322–328 (2005).

71. Khandaker, R. M., Komal, P. P. & Monzur, M. A. Prevalence of *Vibrio spp*. and antibiogram of isolates from shrimp rearing ponds in Bangladesh. *J. Adv. Sci. Res.* **2**, 74-80 (2011).
72. Kim, J. H. et al. Molecular characterization of tetracycline- and quinolone-resistant *Aeromonas salmonicida* isolated in Korea. *J. Vet. Sci.* **12**, 41–48 (2011).
73. Kitiyodom, S., Khemtong, S., Wongtavatchai, J. & Chuanchuen, R. Characterization of antibiotic resistance in *Vibrio* spp. isolated from farmed marine shrimps (*Penaeus monodon*). *FEMS Microbiol. Ecol.* **72**, 219–227 (2010).
74. Korun, J. & Karaca, M. Antibiotic resistance and plasmid profile of *Vibrio alginolyticus* strains isolated from cultured European sea bass (*Dicentrarchus labrax*, L.). *Bull. Vet. Inst. Pulawy* **57**, 173–177 (2013).
75. Kritihi, A., Ouaissa, K., Maychal, A., Oumessaoud, Y., Barakate, M. & Hasnaoui, M. Isolation of a Gram-negative fish pathogen from Moroccan rainbow trout hatchery in winter and its classical characterization. *Int. J. Adv. Enginer. Manag. Sci.* **5**, 57-62 (2019).
76. Kusdarwati, R., Rozi, Dinda, N. D. & Nurjanah, I. Antimicrobial resistance prevalence of *Aeromonas hydrophila* isolates from motile *Aeromonas* septicemia disease. *IOP Conf. Ser.: Earth Environ. Sci.* **137**, 12076 (2018).
77. Laganà, P., Caruso, G., Minutoli, E., Zaccone, R. & Santi, D. Susceptibility to antibiotics of Vibrio spp. and *Photobacterium damsela* ssp. *piscicida* strains isolated from Italian aquaculture farms. *New Microbiol.* **34**, 53–63 (2011).
78. Laith, A. A. et al. Molecular identification and histopathological study of natural *Streptococcus agalactiae* infection in hybrid tilapia (*Oreochromis niloticus*). *Vet. World* **10**, 101–111 (2017).
79. Laith, A. R. & Najiah, M. *Aeromonas hydrophila*: antimicrobial susceptibility and histopathology of isolates from diseased catfish, *Clarias gariepinus* (Burchell). *J. Aquacul. Res. Dev.* **5**, (2014).
80. Lajnef, R., Snoussi, M., Romalde, J. L., Nozha, C. & Hassen, A. Comparative study on the antibiotic susceptibility and plasmid profiles of *Vibrio alginolyticus* strains isolated from four Tunisian marine biotopes. *World J. Microbiol. Biotechnol.* **28**, 3345–3363 (2012).
81. Langaoen, A. F., Manzano, V. J. V., Requiman, E. M. R., Tabardillo, J. M., Maningas, M. B. B. & Calugay, R. J. Antibiotic-resistant bioluminescent *vibrios* from Philippine aquacultured *Chanos chanos* and *Oreochromis niloticus. AACL Bioflux* **11**, 505-515 (2018).
82. Lee, S. W., Najiah, M., Wendy, W. & Nadirah, M. Comparative study on antibiogram of *Vibrio* spp. isolated from diseased postlarval and marketable-sized white leg shrimp (*Litopenaeus vannamei*). *Front. Agric. China* **3**, 446 (2009).
83. Lee, S. W. & Wendy, W. Antibiotic and heavy metal resistance of *Aeromonas hydrophila* and *Edwardsiella tarda* isolated from red hybrid tilapia (*Oreochromis* spp.) coinfected with motile aeromonas septicemia and edwardsiellosis. *Vet. World* **10**, 803–807 (2017).
84. Lee, Y. et al. Occurrence of pathogenic *Vibrio parahaemolyticus* in seafood distribution channels and their antibiotic resistance profiles in S. Korea. *Lett. Appl. Microbiol.* **68**, 128–133 (2019).
85. Leon, J., Avalos, R. & Ponce, M. *Flavobacterium psychrophilum* y su patología en alevines de *Onchorhynchus mykiss* del centro piscícola El Ingenio, Huancayo. *Rev. Peru. Biol.* **15**, 117-124 (2009).
86. Li, Y. & Cai, S.-H. Identification and pathogenicity of *Aeromonas sobria* on tail-rot disease in juvenile tilapia *Oreochromis niloticus*. *Curr. Microbiol.* **62**, 623–627 (2011).
87. Li, Y. W. *et al.* Chronic streptococcosis in Nile tilapia, *Oreochromis niloticus* (L.), caused by *Streptococcus agalactiae*. *J. Fish Dis.* **37**, 757–763 (2014).
88. Lijon, M. B., Kathun, M. M., Islam, A., Khatun, M. M. & Islam, M. A. Detection of multidrug resistance *Aeromonas hydrophila* in farm raised fresh water prawns. *J. Adv. Vet. Anim. Res.* **2**, 469-474 (2015).
89. Liu, J. Y., Li, A. H., Zhou, D. R., Wen, Z. R. & Ye, X. P. Isolation and characterization of

*Edwardsiella ictaluri* strains as pathogens from diseased yellow catfish *Pelteobagrus fulvidraco* (Richardson) cultured in China. *Aquac. Res.* **41**, 1835–1844 (2010).
90. Lo, D. Y., Lee, Y. J., Wang, J. H. & Kuo, H. C. Antimicrobial susceptibility and genetic characterisation of oxytetracycline-resistant *Edwardsiella tarda* isolated from diseased eels. *Vet. Rec.* **175**, 203 (2014).
91. Lü, A. J. et al. Isolation, identification and antimicrobial susceptibility of pathogenic *Aeromonas media* isolated from diseased Koi carp (*Cyprinus carpio* koi). *Iran. J.Fish. Sci.* **15**, 760–774 (2016).
92. Lukkana, M., Jantrakajorn, S. & Wongtavatchai, J. Antimicrobial susceptibility and enrofloxacin resistance of streptococcal bacteria from farmed Nile tilapia, *Oreochromis niloticus* (Linnaeus 1758) in Thailand. *Aquacult. Res.* **47**, 3136–3144 (2016).
93. Luo, X. et al. Isolation, pathogenicity and characterization of a novel bacterial pathogen *Streptococcus uberis* from diseased mandarin fish *Siniperca chuatsi*. *Microb. Pathog.* **107**, 380–389 (2017).
94. Malainine, S. M., Moussaoui, W., Prévost, G., Scheftel, J.-M. & Mimouni, R. Rapid identification of *Vibrio parahaemolyticus* isolated from shellfish, sea water and sediments of the Khnifiss lagoon, Morocco, by MALDI-TOF mass spectrometry. *Lett. App. Microbiol.* **56**, 379–386 (2013).
95. Marhual, N. P., Das, B. K. & Samal, S. K. Characterization of *Vibrio alginolyticus* and *Vibrio parahaemolyticus* isolated from *Penaeus monodon*: antimicrobial resistance, plasmid profiles and random amplification of polymorphic DNA analysis. *African J. Microbiol. Res*. **6**, 4270-4276 (2012).
96. Martínez, B. et al. Differentiation of farmed and wild turbot (*Psetta maxima*): proximate chemical composition, fatty acid profile, trace minerals and antimicrobial resistance of contaminant bacteria. *Food Sci. Technol. Int.* **16**, 435–441 (2010).
97. de Melo, L. M. R. *et al.* Antibiotic resistance of *Vibrio parahaemolyticus* isolated from pond-reared *Litopenaeus vannamei* marketed in Natal, Brazil. *Braz. J. Microbiol.* **42**, 1463–1469 (2011).
98. Meng, F., Kanai, K. & Yoshikoshi, K. Characterization of drug resistance in *Streptococcus parauberis* isolated from Japanese flounder. *Fish Pathol.* **44**, 40–46 (2009).
99. Miranda, C. D., Smith, P., Rojas, R., Contreras-Lynch, S. & Vega, J. M. A. Antimicrobial susceptibility of *Flavobacterium psychrophilum* from Chilean salmon farms and their epidemiological cut-off values using agar dilution and disk diffusion methods. *Front. Microbiol* **7**, (2016).
100. Mo, Z.-Q. et al. Outbreak of *Edwardsiella tarda* infection in farm-cultured giant mottled eel *Anguilla marmorata* in China. *Fish. Sci.* **81**, 899–905 (2015).
101. Mohamad, N. et al. Natural concurrent infection of *Vibrio harveyi* and *V. alginolyticus* in cultured hybrid groupers in Malaysia. *J. Aquat Anim. Health* **31**, 88–96 (2019).
102. Mohammed, H. H. & Arias, C. R. Epidemiology of columnaris disease affecting fishes within the same watershed. *Dis. Aquat. Org.* **109**, 201–211 (2014).
103. Monteiro, S. H. et al. Relationship between antibiotic residues and occurrence of resistant bacteria in Nile tilapia (*Oreochromis niloticus*) cultured in cage-farm. *J. Environ. Sci. Health B* **51**, 817–823 (2016).
104. Najiah, M. et al. Antibiotic resistance and heavy metals tolerance in Gram-negative bacteria from diseased American bullfrog (*Rana catesbeiana*) Cultured in Malaysia. *Agricult. Sci. China* **8**, 1270–1275 (2009).
105. Naviner, M. et al. Antimicrobial resistance of *Aeromonas* spp. isolated from the growth pond to the commercial product in a rainbow trout farm following a flumequine treatment. *Aquaculture* **315**, 236–241 (2011).
106. Ng, C. et al. Microbial water quality and the detection of multidrug resistant *E. coli* and antibiotic resistance genes in aquaculture sites of Singapore. *Mar. Pollut. Bull.* **135**, 475–480 (2018).
107. Nguyen, H. N. K. et al. Molecular characterization of antibiotic resistance in *Pseudomonas* and *Aeromonas* isolates from catfish of the Mekong Delta, Vietnam. *Vet.*

*Microbiol.* **171**, 397–405 (2014).
108. Odeyemi, O. A. & Ahmad, A. Antibiotic resistance profiling and phenotyping of *Aeromonas* species isolated from aquatic sources. *Saudi J. Biol. Sci.* **24**, 65–70 (2017).
109. Ogbonne, F. C., Ukazu, E. R. & Egbe, F. C. Antibiotics resistance pattern and plasmid profiling of *Edwardsiella tarda* isolated from *Heterobranchus longifilis*. *J. Biosci. Med.* **6**, 95 (2018).
110. Oh, E.-G. et al. Antimicrobial resistance of *Vibrio parahaemolyticus* and *Vibrio alginolyticus* strains isolated from farmed fish in Korea from 2005 through 2007. *J. Food Prot.* **74**, 380–386 (2011).
111. Orozova, P., Chikova, V. & Najdenski, H. Antibiotic resistance of pathogenic for fish isolates of *Aeromonas* spp. *Bulg. J. Agric. Sci.* **16,** 376-386 (2010).
112. Osman, K. M. et al. Characterization and susceptibility of streptococci and enterococci isolated from Nile tilapia (*Oreochromis niloticus*) showing septicaemia in aquaculture and wild sites in Egypt. *BMC Vet. Res.* **13**, 357 (2017).
113. Otterlei, A. et al. Phenotypic and genetic characterization of *Piscirickettsia salmonis* from Chilean and Canadian salmonids. *BMC Vet. Res.* **12**, 55 (2016).
114. Özer, S. et al. Genetic diversity and antimicrobial susceptibility of motile aeromonads isolated from rainbow trout (*Oncorhynchus mykiss*, Walbaum) farms. *J. App. Ichthyol.* **25**, 195–200 (2009).
115. Park, Y.-K. et al. Antibiotic susceptibility and resistance of *Streptococcus iniae* and *Streptococcus parauberis* isolated from olive flounder (*Paralichthys olivaceus*). *Vet. Microbiol.* **136**, 76–81 (2009).
116. Patil, H. J. et al. Evidence of increased antibiotic resistance in phylogenetically-diverse *Aeromonas* isolates from semi-intensive fish ponds treated with antibiotics. *Front Microbiol* **7**, (2016).
117. Pedonese, F. et al. Occurrence and antimicrobial susceptibility of aeromonads from maricultured gilthead seabream (*Sparus aurata*). in *Animal farming and environmental interactions in the Mediterranean region* (eds. Casasús, I., Rogošiç, J., Rosati, A., Štokoviç, I. & Gabiña, D.) 205–209 (Wageningen Academic Publishers, 2012). doi:10.3920/978-90-8686-741-7_25
118. Perretta, A., Antúnez, K. & Zunino, P. Phenotypic, molecular and pathological characterization of motile aeromonads isolated from diseased fishes cultured in Uruguay. *J. Fish Dis.* **41**, 1559–1569 (2018).
119. Rahman, M. M. & Hossain, M. N. Antibiotic and herbal sensitivity of some *Aeromonas* sp. isolates collected from diseased carp fishes. *Progress. Agric.* **21**, 117–129 (2010).
120. Rahman, M. et al. Molecular Identification of multiple antibiotic resistant fish pathogenic *Enterococcus faecalis* and their control by medicinal herbs. *Sci. Rep.* **7**, 3747 (2017).
121. Ramesh, D. & Souissi, S. Antibiotic resistance and virulence traits of bacterial pathogens from infected freshwater fish, Labeo rohita. *Microb. Pathog.* **116**, 113–119 (2018).
122. Ransangan, J., Imm, L. K. L., Lal, T. M. & Sade, A. Phenotypic characterization and antibiotic susceptibility of *Vibrio* spp. isolated from aquaculture waters on the west coast of Sabah, Malaysia. *J. Microbiol. Immunol. Inf.* **49**, 591–594 (2016).
123. Ransangan, J. & Mustafa, S. Identification of *Vibrio harveyi* isolated from diseased Asian seabass *Lates calcarifer* by use of 16S ribosomal DNA sequencing. *J. Aquat. Anim. Health* **21**, 150–155 (2009).
124. Rebouças, R. H. et al. Antimicrobial resistance profile of *Vibrio* species isolated from marine shrimp farming environments (*Litopenaeus vannamei*) at Ceará, Brazil. *Environ. Res.* **111**, 21–24 (2011).
125. Resende, J. A. et al. Multidrug-resistance and toxic metal tolerance of medically important bacteria isolated from an aquaculture system. *Microbes Environ* **27**, 449–455 (2012).
126. Revina, O., Latvia Univ. of Agriculture, J. (Latvia), Avsejenko, J., Cirule, D. & Valdovska, A. Antimicrobial resistance of *Aeromonas* spp. isolated from the sea trout (*Salmo trutta* L.) in Latvia. in *Research for Rural Development. International Scientific Conference*

*Proceedings (Latvia)* (Latvia University of Agriculture, 2017).
127. Reyes, A., Bullanday, M. J. C. & Fajardo., L. J. Antibiotics susceptibility of *Streptococcus agalactiae* isolated from tilapia pond water in Lubao, Pampanga, Philippines. *Int. J. Biol. Pharmacy Allied Sci.* **7**, 1702–1716 (2018).
128. Rocha, R. D. S., de Sousa, O. V. & Vieira, R. H. S. D. F. Multidrug-resistant *Vibrio* associated with an estuary affected by shrimp farming in Northeastern Brazil. *Mar. Pollut. Bull.* **105**, 337–340 (2016).
129. Rogge, M. L. et al. Comparison of Vietnamese and US isolates of *Edwardsiella ictaluri*. *Dis. Aquat. Org.* **106**, 17–29 (2013).
130. Ruzauskas, M. et al. Composition and antimicrobial resistance profile of Gram-negative microbiota prevalent in aquacultured fish. *J. Food Saf.* **38**, e12447 (2018).
131. Sahoo, P. K. et al. Detection of goldfish haematopoietic necrosis herpes virus (Cyprinid herpesvirus-2) with multi-drug resistant *Aeromonas hydrophila* infection in goldfish: First evidence of any viral disease outbreak in ornamental freshwater aquaculture farms in India. *Acta Trop.* **161**, 8–17 (2016).
132. Saidi, N., Lagha, R., Abdallah, F. B., Rokbani, K. B. & Bakhrouf, A. Slime producing, heavy metals and antibiotic resistance in *Aeromonas hydrophila* isolated in Tunisia. *African J. Microbiol. Res.* **7**, 5697-5708 (2013).
133. Saifedden, G., Farinazleen, G., Nor-Khaizura, A., Kayali, A. Y., Nakaguchi, Y., Nishibuchi, M. & Son, R. Antibiotic susceptibility profile of *Vibrio parahaemolyticus* isolated from shrimp in Selangor, Malaysia. *Int. Food Res. J.* **23**, 2732-2736 (2016).
134. Sakala, T. *Identification and antibiogram profiles of bacteria associated with diseased Oreochromis niloticus in lake Kariba, Zambia.* Master dissertation, Sokoine University of Agriculture Morogoro, Tanzania (2017).
135. Samal, S. K., Das, B. K. & Pal, B. B. Isolation, biochemical characterization, antibiotic susceptibility study of *Aeromonas hydrophila* isolated from freshwater fish. *Int. J. Curr. Microbiol. App. Sci.* **3**, 259-267 (2014).
136. Sarder, H. et al. Prevalence and antibiotic susceptibility of *Aeromonas hydrophila* isolated from freshwater fishes. *J. Fish.* **4**, 411–419 (2016).
137. Scarano, C. et al. Antibiotic resistance of *Aeromonas* ssp. strains isolated from *Sparus aurata* reared in Italian mariculture farms. *Int. J. Food Microbiol.* **284**, 91–97 (2018).
138. Scarano, C. et al. Antibiotic resistance of *Vibrio* species isolated from *Sparus aurata* reared in Italian mariculture. *New Microbiol.* **37**, 329–337 (2014).
139. Shakerian, A., Barton, M. D., Akinbowale, O. L. & Khamesipour, F. Antimicrobial resistance profile and resistance genes of *Vibrio* species isolated from giant freshwater prawn (*Macrobrachium rosenbergii*) raised in Iran. *J. Hellenic Vet. Med. Soc.* **68**, 79–88 (2017).
140. Shao-wu, L., Di, W., Hong-bai, L. & Tong-yan, L. Molecular typing of *Aeromonas hydrophila* isolated from common carp in Northeast China. *J. Northeast Agric. Univ. (English Edition)* **20**, 30–36 (2013).
141. Shahzad, A., Iahtasham, K., Qurban, A., Ullah, K. S., Sharif, M. Z. & Shamim, A. Isolation and characterisation of *Aeromonas sobria* in *Catla catla* (Thaila) affected with hemorrhagic septicaemia. *Bull. Eur. Ass. Fish Pathol.* **34**, 35-42 (2014).
142. Sierralta, V. et al. Patología e identificación de *Yersinia ruckeri* en trucha arco iris (*Oncorhynchus mykiss*) en piscigranjas de Junín, Perú. *Revista AquaTIC* **0**, (2016).
143. Silvester, R., Alexander, D. & Ammanamveetil, M. H. A. Prevalence, antibiotic resistance, virulence and plasmid profiles of *Vibrio parahaemolyticus* from a tropical estuary and adjoining traditional prawn farm along the southwest coast of India. *Ann. Microbiol.* **65**, 2141–2149 (2015).
144. Singh, B., Tyagi, A., Thammegowda, N. K. B. & Ansal, M. D. Prevalence and antimicrobial resistance of vibrios of human health significance in inland saline aquaculture areas. *Aquac. Res.* **49**, 2166–2174 (2018).
145. Soto, E. et al. *Edwardsiella ictaluri* as the causative agent of mortality in cultured Nile tilapia. *J. Aquat. Anim. Health* **24**, 81–90 (2012).
146. Soto, E. et al. Characterization of isolates of *Streptococcus agalactiae* from diseased

farmed and wild marine fish from the U.S. Gulf Coast, Latin America, and Thailand. *J. Aquat. Anim. Health* **27**, 123–134 (2015).
147. Soto-Rodriguez, S. A. et al. Virulence of the fish pathogen *Aeromonas dhakensis*: genes involved, characterization and histopathology of experimentally infected hybrid tilapia. *Dis. Aquat. Org.* **129**, 107–116 (2018).
148. Sperling, L., Alter, T. & Huehn, S. Prevalence and antimicrobial resistance of *Vibrio* spp. in retail and farm shrimps in Ecuador. *J. Food Prot.* **78**, 2089–2092 (2015).
149. Sreedharan, K., Philip, R. & Singh, I. S. B. Virulence potential and antibiotic susceptibility pattern of motile aeromonads associated with freshwater ornamental fish culture systems: a possible threat to public health. *Braz. J. Microbiol.* **43**, 754–765 (2012).
150. Stalin, N. & Srinivasan, P. Molecular characterization of antibiotic resistant *Vibrio harveyi* isolated from shrimp aquaculture environment in the south east coast of India. *Microb. Pathog.* **97**, 110–118 (2016).
151. Su, H.-C. et al. Occurrence of antibiotic resistance and characterization of resistance genes and integrons in Enterobacteriaceae isolated from integrated fish farms in South China. *J. Environ. Monit.* **13**, 3229–3236 (2011).
152. Stratev, D., Vashin, I. & Daskalov, H. Antimicrobial resistance of b-haemolytic *Aeromonas hydrophila* strains isolated from rainbow trout (*Oncorhynchus mykiss*). *Bulg. J. Vet. Med.* **16,** 289-296 (2013).
153. Stratev, D., Daskalov, H., Vashin, I. Characterisation and determination of antimicrobial resistance of β-haemolytic *Aeromonas* spp. isolated from common carp (*Cyprinus carpio* L.). *Revue Méd. Vét.* **166**, 54-61 (2015).
154. Suanyuk, N. et al. Mortality and pathology of hybrid catfish, *Clarias macrocephalus* (Günther) × *Clarias gariepinus* (Burchell), associated with *Edwardsiella ictaluri* infection in southern Thailand. *J. Fish Dis.* **37**, 385–395 (2014).
155. Syrova, E. et al. Antibiotic resistance and virulence factors in mesophilic *Aeromonas* spp. from Czech carp fisheries. *J. Appl. Microbiol.* (2018). doi:10.1111/jam.14075
156. Tabo, N. A., Ramirez, V. B., Tabo, H. A. L. & Gloriani, N. G. Occurrence and antimicrobial resistance of pathogenic *Vibrios* isolated from green mussel, *Perna viridis* L. 1758 in Bacoor Bay, Cavite, Philippines. *Acta Med. Philipp.* **49**, 39–44 (2015).
157. Tavares, G. C. *et al.* Disease outbreaks in farmed Amazon catfish (*Leiarius marmoratus x Pseudoplatystoma corruscans*) caused by *Streptococcus agalactiae, S. iniae*, *and S. dysgalactiae*. *Aquaculture* **495**, 384–392 (2018).
158. Tiamiyu, A. M., Soladoye, M. O., Adegboyega, T. T. & Adetona, M. O. Occurrence and antibiotic sensitivity of bacterial strains isolated from Nile tilapia, *Oreochromis niloticus* obtained in Ibadan, Southwest Nigeria. *J. Biosci. Med.* **3**, 19 (2015).
159. Türe, M. & Alp, H. Identification of bacterial pathogens and determination of their antibacterial resistance profiles in some cultured fish in Turkey. *J. Vet. Res.* **60**, 141–146 (2016).
160. Ture, M., Altinok, I. & Alp, H. Effects of cage farming on antimicrobial and heavy metal resistance of *Escherichia coli, Enterococcus faecium*, and *Lactococcus garvieae*. *Microb. Drug Resis.* **24**, 1422–1430 (2018).
161. Ture, M. & Boran, H. Phenotypic and genotypic antimicrobial resistance of *Lactococcus sp.* strains isolated from rainbow trout (*Oncorhynchus mykiss*). *Bull. Vet. Inst. Pulawy* **59**, 37–42 (2015).
162. Uhland, F. C. Characterization of antimicrobial resistance in *Aeromonas* and *Vibrio* isolated in Canada from fish and seafood. (2011).
163. Ulkhaq, M. F. & Lusiastuti, A. M. Resistance test on *Aeromonas hydrophila* isolated from African catfish (*Clarias gariepinus*) against some antibiotics groups. *Microbiol. Indonesia* **11**, 5 (2017).
164. Valdebenito, S. & Avendaño-Herrera, R. Phenotypic, serological and genetic characterization of *Flavobacterium psychrophilum* strains isolated from salmonids in Chile. *J. Fish Dis.* **32**, 321–333 (2009).
165. Valdes, N. et al. Draft genome sequence of the Chilean isolate *Aeromonas salmonicida*

strain CBA100. *FEMS Microbiol. Lett.* **362**, (2015).
166. Vega-Sánchez, V. et al. Phenotypical characteristics, genetic identification, and antimicrobial sensitivity of *Aeromonas* species isolated from farmed rainbow trout (*Onchorynchus mykiss*) in Mexico. *Acta Trop.* **130**, 76–79 (2014).
167. Wamala, S. P. et al. Occurrence and antibiotic susceptibility of fish bacteria isolated from *Oreochromis niloticus* (Nile tilapia) and *Clarias gariepinus* (African catfish) in Uganda. *Fish Aquatic Sci.* **21**, 6 (2018).
168. Wang, R. X., Wang, J. Y., Sun, Y. C., Yang, B. L., & Wang, A. L. Antibiotic resistance monitoring in *Vibrio* spp. isolated from rearing environment and intestines of abalone *Haliotis diversicolor. Mar. Pollut. Bull.* **101**, 701–706 (2015).
169. Wang, K. et al. Isolation and characterisation of *Streptococcus agalactiae* from Nile tilapia *Oreochromis niloticus* in China. . *African J. Microbiol. Res*. **7**, 317-323 (2013).
170. Wei, L. S., Mustakim, M. T., Azlina, N., Zulhisyam, A. K., An'amt, M. N., Wee, W. & Huang, N. M. Antibiotic and heavy metal resistance of *Aeromonas* spp. isolated from diseased red hybrid tilapia (*Oreochromis* sp.). *Ann. Res. Rev. Biol.* **6**, 264-269 (2015).
171. Wei, L. S. et al. Antibiogram and plasmid profiling from *Edwardsiella tarda* isolated from freshwater fish from the East coast of Malaysia. *J. Sustain. Sci. Manag.* **6**, 19–27 (2011).
172. Wimalasena, S. H. M. P., Pathirana, H. N. K. S., de Silva, B. C. J., Hossain, S., Sugaya, E., Nakai, T. & Heo, G-J. Antibiotic resistance and virulence-associated gene profiles of *Edwardsiella tarda* isolated from cultured fish in Japan. *Turk. J. Fish Aquat. Sci.* **19,** 141-148 (2019).
173. Xiao, J. et al. Isolation and identification of fish pathogen *Edwardsiella tarda* from mariculture in China. *Aquac. Res.* **40**, 13–17 (2008).
174. Yang, Q. et al. Multidrug-resistant *Aeromonas veronii* recovered from channel catfish (*Ictalurus punctatus*) in China: prevalence and mechanisms of fluoroquinolone resistance. *Microb. Drug Resist.* **23**, 473–479 (2017).
175. Yang, Y. et al. Antibiotic susceptibility and molecular characterization of *Aeromonas hydrophila* from grass carp. *J. Food Saf.* **38**, e12393 (2018).
176. Yano, Y. et al. Prevalence and antimicrobial susceptibility of *Vibrio* species related to food safety isolated from shrimp cultured at inland ponds in Thailand. *Food Control* **38**, 30–36 (2014).
177. Yano, Y. et al. Occurrence, molecular characterization, and antimicrobial susceptibility of *Aeromonas* spp. in marine species of shrimps cultured at inland low salinity ponds. *Food Microbiol.* **47**, 21–27 (2015).
178. Ye, Y. et al. Resistance characterization, virulence factors, and ERIC-PCR fingerprinting of *Aeromonas veronii* strains isolated from diseased *Trionyx sinensis*. *Foodborne Pathog. Dis.* **9**, 1053–1055 (2012).
179. Yi, S-W., Kim, D-C., You, M-J., Kim, B-S., Kim, W. & Shin, G-W. Antibiotic and heavy-metal resistance in motile *Aeromonas* strains isolated from fish. *African J. Microbiol. Res*. **8**, 1793-1797 (2014).
180. Yu, J.-H., Han, J. J., Park, K. S., Park, K. H. & Park, S. W. *Edwardsiella tarda* infection in Korean catfish, *Silurus asotus*, in a Korean fish farm. *Aquac. Res.* **41**, 19–26 (2009).
181. Yue, X., Liu, B., Xiang, J. & Jia, J. Identification and characterization of the pathogenic effect of a *Vibrio parahaemolyticus*-related bacterium isolated from clam *Meretrix meretrix* with mass mortality. *J. Invertebr. Pathol.* **103**, 109–115 (2010).
182. Zaman, B. S. et al. Bacterial flora of koi (*Anabas testudineus*) harvested from ponds and their Antibiogram. *Microb. Health* **2**, 8–11 (2013).
183. Zhang, Z. et al. The pathogenic and antimicrobial characteristics of an emerging *Streptococcus agalactiae* serotype IX in Tilapia. *Microb. Pathog.* **122**, 39–45 (2018).
184. Zhao, S. et al. Antimicrobial resistance and pulsed-field gel electrophoresis typing of *Vibrio parahaemolyticus* isolated from shrimp mariculture environment along the east coast of China. *Mar. Pollut. Bull.* **136**, 164–170 (2018).
185. Zheng, W., Cao, H. & Yang, X. Grass carp (*Ctenopharyngodon idellus*) infected with multiple strains of *Aeromonas hydrophila. African J. Microbiol. Res*. **6**, 4512-4520 (2012).

186. Zhu, Z. M., Dong, C. F., Weng, S. P. & He, J. G. The high prevalence of pathogenic *Vibrio harveyi* with multiple antibiotic resistance in scale drop and muscle necrosis disease of the hybrid grouper, *Epinephelus fuscoguttatus* (♀) × *E. lanceolatus* (♂), in China. *J. Fish Dis.* **41**, 589–601 (2018).
187. Zouiten, A. et al. Designation of pathogenic resistant bacteria in the *Sparus aurata* sea collected in Tunisia coastlines: Correlation with high performance liquid chromatography-tandem mass spectrometry analysis of antibiotics. *Microb. Pathog.* **106**, 3–8 (2017).
