# Supplementary material for: Aquaculture at the crossroads of global warming and antimicrobial resistance
Source: Nat Commun. 2020 Apr 20;11:1870. doi: 10.1038/s41467-020-15735-6 (PMC7170852; doi:10.1038/s41467-020-15735-6)
Supplement: Supplementary file 6 — Reporting Summary [file 41467_2020_15735_MOESM6_ESM.pdf]

nature research

Corresponding author(s): Miriam Reverter & Rodolphe Gozlan

Last updated by author(s): 26/02/2020

Reporting Summary

Nature Research wishes to improve the reproducibility of the work that we publish. This form provides structure for consistency and transparency in reporting. For further information on Nature Research policies, see Authors & Referees and the Editorial Policy Checklist .

Statistics

For all statistical analyses, confirm that the following items are present in the figure legend, table legend, main text, or Methods section.

n/a	Confirmed	
☐	☒	The exact sample size (*n*) for each experimental group/condition, given as a discrete number and unit of measurement
☐	☒	A statement on whether measurements were taken from distinct samples or whether the same sample was measured repeatedly
☐	☒	The statistical test(s) used AND whether they are one- or two-sided<br>*Only common tests should be described solely by name; describe more complex techniques in the Methods section.*
☐	☒	A description of all covariates tested
☐	☒	A description of any assumptions or corrections, such as tests of normality and adjustment for multiple comparisons
☐	☒	A full description of the statistical parameters including central tendency (e.g. means) or other basic estimates (e.g. regression coefficient) AND variation (e.g. standard deviation) or associated estimates of uncertainty (e.g. confidence intervals)
☐	☒	For null hypothesis testing, the test statistic (e.g. *F*, *t*, *r*) with confidence intervals, effect sizes, degrees of freedom and *P* value noted<br>*Give P values as exact values whenever suitable.*
☒	☐	For Bayesian analysis, information on the choice of priors and Markov chain Monte Carlo settings
☐	☒	For hierarchical and complex designs, identification of the appropriate level for tests and full reporting of outcomes
☒	☐	Estimates of effect sizes (e.g. Cohen's *d*, Pearson's *r*), indicating how they were calculated

Our web collection on statistics for biologists contains articles on many of the points above.

Software and code

Policy information about availability of computer code

Data collection	Web of Science Core collection and Google Scholar were used to systematically search articles and theses on the subjects of interest. We performed two independent searches with the following keyword combinations: 1) (aquaculture* OR farm* OR rear*) AND (fish OR shrimp OR shellfish) AND (mortality OR outbreak OR infection) AND (Aeromonas OR Edwardsiella OR Flavobacterium OR Streptococc* OR Vibrio OR Yersinia OR KHV OR Ostreid HV) and 2) (antimicrobial or antibiotic) AND (resistance OR susceptibil*) AND (aquaculture OR fish OR shrimp OR shellfish).
Data analysis	All data were analysed using R version 3.6.0.<br>Function lmer from package lme4 (v1.1-21) was used to perform the mixed effect models.<br>Function model.sel from package MuMIn (v1.43.6) was used to select the models with the highest Akaike weight.<br>Function confint from package lme4 (v1.1-21) was used to calculate confidence intervals of the explanatory variables of the selected models.<br>Function cor.test from package stats (v3.6.0) was used to correlated MAR indices with environmental and socio-economic indicators.<br>All plots were performed using the package ggplot2 (v3.2.0).<br>The MAR indices figure was performed using QGIS3 (version 3.4.3.).

For manuscripts utilizing custom algorithms or software that are central to the research but not yet described in published literature, software must be made available to editors/reviewers. We strongly encourage code deposition in a community repository (e.g. GitHub). See the Nature Research guidelines for submitting code & software for further information.

Data

Policy information about availability of data

All manuscripts must include a data availability statement. This statement should provide the following information, where applicable:

- Accession codes, unique identifiers, or web links for publicly available datasets
- A list of figures that have associated raw data
- A description of any restrictions on data availability

All data generated from this study (meta-data used to study the mortalities of infected aquatic animals in relationship to temperature and meta-data used to calculate the aquaculture-derived MAR) is available on the public repository DRYAD (temporary link where data can be downloaded, https://datadryad.org/stash/share/JK183pVtScGkNSiRyj4B9LMpRDQzVifGDgEVF5pUR_o)
Data on environmental and socio-economic indicators used for our correlation studies with the multiple-antibiotic resistance index (MAR) issued from aquaculture-related bacteria was collected from these sources:
- Worldbank Open Database (https://data.worldbank.org) - Gross Domestic Product (GDP) per capita, Human Development Index (HDI), population, prevalence of undernourishment, % of people using at least basic sanitation services and % of population using at least basic water services data.
- FAOSTAT webpage (http://www.fao.org/faostat) - Pesticide use, animal production, livestock density and animal trade data.
- WAPI Aquaculture Production module developed by the FAO (http://www.fao.org/fishery/statistics/software/wapi/en) - aquaculture production data
- Environmental Performance Index (EPI- webpage (https://epi.envirocenter.yale.edu) - EPI data
- ResistanceMap.cddep.org, owned and operated by the Center for Disease Dynamics, Economics & Policy, Inc. (CDDEP) - clinical antibiotic use data and the antibiotic resistance (number of isolates tested and % of resistance) of clinical Escherichia coli to aminoglycosides, 3rd generation cephalosporins and fluoroquinolones.
- HSBC climate vulnerability data (cited in Paun et al. 2018).
- Climate change knowledge portal (https://climateknowledgeportal.worldbank.org) - regional average temperature data (1991 - 2016) using the GPS coordinates of the studies where the data for MAR calculation was obtained.

The authors declare that all data supporting the findings of this study are available within the paper and its supplementary information files.

Field-specific reporting

Please select the one below that is the best fit for your research. If you are not sure, read the appropriate sections before making your selection.

☐ Life sciences ☐ Behavioural & social sciences ☒ Ecological, evolutionary & environmental sciences

For a reference copy of the document with all sections, see nature.com/documents/nr-reporting-summary-flat.pdf

Ecological, evolutionary & environmental sciences study design

All studies must disclose on these points even when the disclosure is negative.

Study description	We conducted a double meta-analysis (481 articles) to explore how global warming and antimicrobial resistance (AMR) impacts aquaculture. The first part of the article explored the relationship between temperature and mortality of aquatic animals infected with common aquaculture pathogens (7 bacterial genera and 2 viruses). We extracted a total of 651 observations from 294 studies and we performed nested mixed effect models to select for the best model fit (fixed effects: temperature, life stage, mode of infection, infective dose (log-transformed), and interaction between mode of infection and log(dose), random effects: host and pathogen taxonomy). In the second part of the study we calculated a Multi-Antibiotic Resistance index (MAR) of aquaculture-related bacteria (11,274 strains) for 40 countries (at least 30 strains/country) and we correlated them to several environmental and socio-economic indicators (climate vulnerability index, temperature, environmental performance index, GDP per capita, undernourishement, human development index, pesticide use, aquaculture production, livestock production, animal trade, livestock density, total use of clinical antibiotics, MAR of clinical bacteria, basic sanitation services and basic water services).
Research sample	For the first part of the study we considered all types of diseased cultured aquatic animals (molluscs, arthropods, fish) infected with major aquaculture pathogens. Studies with animals with different life stages (adults, juveniles) were included in the dataset, and the factor life stage was treated as a fixed effect in the models. Aquaculture pathogens were chosen according to their importance in aquaculture and the literature (reporting temperatures) available.<br>For the calculation of aquaculture-related MAR indices we used all articles found based on the criteria defined that reported antibiotic resistance of bacterial strains identified to genus level (in order to be able to disregard natural antibiotic susceptibilities). Only pathogenic bacteria for the animals were included (e.g. Escherichia coli or other bacteria that could have come from contamination sources were excluded). MAR index was calculated for countries for which we obtained the antibiotic susceptibilities for at least 30 strains.
Sampling strategy	Sampling size was not pre-determined. A systematic review was performed and all results that matched the established criteria were included in the article.
Data collection	Data collection was obtained from the literature following the PRISMA guidelines and was performed by Miriam Reverter.
Timing and spatial scale	We started collecting the published data in October 2018 up to 1st March 2019. Since AMR changes over time, we only retained articles on this subject published within the last 10 years (2009 - 2019).

Data exclusions	Pseudomonas strains were excluded from our antibiotic resistance dataset to avoid bias since they are known to present numerous intrinsic resistances.
Reproducibility	No experiments were performed in this study.
Randomization	Since this is a meta-analytic study we did not use experimental randomization. We included however several co-variates in our model (mortality vs. temperature) to account for other sources of data variability (life stage, mode of infection, infective dose) than the one that interested us (temperature).
Blinding	Blinding was not relevant because there was no experimental setup were the observed could have influenced the results. All results were collected and then analysed without a priori and taking into consideration all variables thought to possibly influence the outcome.

Did the study involve field work? ☐ Yes ☒ No

Reporting for specific materials, systems and methods

We require information from authors about some types of materials, experimental systems and methods used in many studies. Here, indicate whether each material, system or method listed is relevant to your study. If you are not sure if a list item applies to your research, read the appropriate section before selecting a response.

Materials & experimental systems

n/a	Involved in the study
☒	☐ Antibodies
☒	☐ Eukaryotic cell lines
☒	☐ Palaeontology
☒	☐ Animals and other organisms
☒	☐ Human research participants
☒	☐ Clinical data

Methods

n/a	Involved in the study
☒	☐ ChIP-seq
☒	☐ Flow cytometry
☒	☐ MRI-based neuroimaging
